# Unprescribed cannabinoids and multiple sclerosis: a multicenter, cross-sectional, epidemiological study in Lombardy, Italy

**DOI:** 10.1007/s00415-024-12472-4

**Published:** 2024-06-07

**Authors:** Riccardo Giossi, Martina Mercenari, Massimo Filippi, Chiara Zanetta, Carlo Giuseppe Antozzi, Laura Brambilla, Paolo Confalonieri, Sebastiano Giuseppe Crisafulli, Eugenia Tomas Roldan, Pietro Annovazzi, Marta Zaffira Conti, Caterina Barrilà, Marco Ronzoni, Monica Grobberio, Attilio Negri, Stefan Gustavsen, Valentina Torri Clerici

**Affiliations:** 1 Neuroimmunology and Neuromuscular Diseases Unit, Fondazione IRCCS Istituto Neurologico Carlo Besta, Via Celoria, 11, 20133 Milan, Italy; 2 Poison Control Center and Clinical Pharmacology Unit, ASST Grande Ospedale Metropolitano Niguarda, Piazza Ospedale Maggiore, 3, 20162 Milan, Italy; 3 Neurology Department, School of Medicine and Surgery and Milan Centre for Neuroscience (NeuroMI), University of Milano-Bicocca, Milan, Italy; 4 Neurology Unit and MS Center, IRCCS San Raffaele Scientific Institute, Milan, Italy; 5 Neuroimaging Research Unit, Division of Neuroscience, IRCCS San Raffaele Scientific Institute, Milan, Italy; 6 Vita-Salute San Raffaele University, Milan, Italy; 7 Neurorehabilitation Unit, IRCCS San Raffaele Scientific Institute, Milan, Italy; 8 Neurophysiology Service, IRCCS San Raffaele Scientific Institute, Milan, Italy; 9 Neurology Unit, Neurorehabilitation Unit, and Multiple Sclerosis Center, Division of Neuroscience, IRCCS San Raffaele Scientific Institute, Via Olgettina, 60, 20132 Milan, Italy; 10 Multiple Sclerosis Center, Neurology II Unit, ASST Valle Olona, Gallarate Hospital, Gallarate, Italy; 11 Department of Neurology, ASST Papa Giovanni XXIII, Bergamo, Italy; 12 Neurology Unit, ASST Rhodense, Ospedale Garbagnate Milanese, Garbagnate Milanese, Italy; 13 Clinical Neuropsychology Lab, Neurology Department and Clinical Psychology Unit, ASST Lariana, Como, Italy; 14 SC SerD Territoriale-SS SerD Boifava, ASST Santi Paolo e Carlo, Milan, Italy; 15 Department of Neurology, Danish Multiple Sclerosis Center, Copenhagen University Hospital-Rigshospitalet, Glostrup, Denmark

**Keywords:** Cannabis, Multiple sclerosis, Epidemiology, Cross-sectional

## Abstract

**Introduction:**

Cannabinoids are approved for spasticity and pain in multiple sclerosis (MS). In 2017 the prevalence of current users in the Italian general population was 10.2%, while data on Italian MS patients are limited.

**Methods:**

From March 2022 to February 2023, we conducted a multicenter, cross-sectional study. Adult MS patients completed an anonymous online survey. The primary outcome was the estimated prevalence of unprescribed cannabis current use. Cannabis use patterns and associations with clinical and socio-demographical variables were investigated. The binomial method was used to estimate 95% confidence interval (95% CI) for primary outcome.

**Results:**

5620 patients were invited and 2024 (36.0%) were included (mean age 45.2 years, females 64.5%). Relapsing remitting form was the most frequent (77.3%). Median expanded disability status scale (EDSS) was 2.0. The proportion of current users was 15.5% (95% CI 13.9–17.1) and 36.4% of them disclosed to their physician their unprescribed cannabis use. 15.0% patients were former users while 69.5% never used cannabis. Current users more frequently reported a medical use (i.e., current medical users) compared to former users (*p* < 0.001). 41.1% of never users would use cannabis if it was legal. Young age, being male, and a free marital status were associated with current use. Current medical users had higher disability, spasticity and pain, reduced quality of life, concomitant neurological/psychiatric drugs and analgesics use. Unprescribed cannabis appeared relatively safe, with limited addiction risk, and reported clinical benefits, including concomitant medications reduction.

**Conclusion:**

Unprescribed cannabis use is common in patients with MS in Italy, with observed prevalence seemingly superior to the general population, often intended for medical use and without the disclosure to the treating physician, although with potential clinical benefits.

**Supplementary Information:**

The online version contains supplementary material available at 10.1007/s00415-024-12472-4.

## Introduction

Multiple sclerosis (MS) is an inflammatory, demyelinating, neurodegenerative disease of the central nervous system and an important cause of disability [1]. Different forms exist in MS, since progression sutained by neurodegenerative damage superimposes to relapsing inflammatory events. Thus, MS is classically divided into relapsing remitting (RRMS), secondary progressive (SPMS), and primary progressive (PPMS) forms with different disease modifying therapies (DMT) used to contrast clinical evolution. To manage MS-related symptoms, patients often need several drugs other than DMT and *Cannabis sativa* compounds are indicated to treat symptoms of spasticity [2–5]. The two most represented cannabinoids in *Cannabis sativa* are tetrahydrocannabinol (THC) and cannabidiol (CBD). THC is a psychoactive, analgesic, and myorelaxant molecule while CBD exerts relaxant and tranquilizer effects as well as modulatory effects on THC [6–8]. Global research interest in cannabinoids is increasing and Italian legislation already indicated cannabis-based medicine for spasticity and neuropathic pain [9]. Besides prescriptions cannabinoids, the evidence from literature suggests that the use of illegal or unprescribed cannabis is common in MS patients for medical purposes [10–14]. The estimated Italian prevalence of cannabis ever users between 15 and 64 years old is about 33% [15], and in 2017 the prevalence of current users in the general population was 10.2% [16]. Data on cannabis use in the Italian MS population are limited [17]. Also, sociodemographic variables associated with cannabis use and potential effectiveness and safety outcomes have been sparsely investigated in literature and data are lacking in the Italian population. For these reasons, we wanted to evaluate the attitude to cannabinoids consumption in a cohort of Italian patients.

## Materials and methods

### Study design and population

This is a multicenter, cross-sectional, epidemiological study conducted in six MS centers in Lombardy, Italy (Supplementary Material S1). From March 2022 we invited patients with any clinical form of MS or clinically isolated syndrome (CIS), 18 years of age or older, able to give valid informed consent, and followed at participating centers to complete an anonymous online survey realized using REDCap (Research Electronic Data Capture), hosted by IRCCS Istituto Neurologico Carlo Besta, which was accessible via home systems and mobile devices (e.g., smartphone, tablet, personal computer) through a direct link or a QRcode. Patients were invited by mailing lists and by direct invitation during hospital admissions or outpatient visits. An email recall was performed between October and December 2022. Data collection remained open until February 2023. The study was performed in accordance with ethical standards laid down in the 1964 Declaration of Helsinki and its later amendments and was approved by participating centers Institutional Review Boards (IRBs); patients provided informed consent in a dedicated section of the online survey. The STrengthening the Reporting of OBservational studies in Epidemiology (STROBE) statement was followed for the realization of this study (Supplementary Material S2).

### Collected variables and study outcomes

The primary outcome was the estimated prevalence of unprescribed cannabis current users among patients with MS or CIS. Patients were classified as “current users” if they consumed any form of cannabis or cannabinoids without medical prescription, illegal cannabis included, in the last 12 months. Patients who previously consumed cannabis at any time during life but not in the last 12 months were defined as “former users”, while those who never assumed cannabis were defined as “never users”. Secondary objectives were to identify patterns and features of cannabis use; to explore potential associations between cannabis use, sociodemographic and clinical variables, including the last expanded disability status scale (EDSS) score (as reported in their last inpatient or outpatient visit and assessed by a neurologist, if available), the patient determined disease step (PDDS), current medications, and quality of life (QoL); to evaluate potential risk factors for cannabis use in MS patients; to evaluate possible beneficial or adverse effects. Cannabis use-related variables included motive (recreational, medical, or both), frequency, average dose consumption, disclosure of use to the physician, effects of the substances, tobacco or alcohol use. The 29-questions Multiple Sclerosis Quality of Life (MSQoL-29) and the Hospital Anxiety and Depression Scale (HADS) questionnaires were also included in the online survey. MSQoL-29 is constituted by different subscales and items with scores ranging 0–100 and higher scores indicating better QoL [18]. HADS is a measure of anxiety and depression symptoms divided into two subscales with scores ranging 0–21 and higher scores indicating more severe symptoms. A cut-off score > 7 points indicates the presence of possible anxiety or depression [19]. Due to the coincidence of the study period to the COVID-19 pandemic, information on the impact of COVID-19 on cannabis consumption was collected.

### Sample size, database cleaning, and statistical analyses

In order to address the primary objective of the study, given the results of a recent observational study in Denmark reporting a prevalence of current users of 21% among MS patients and the increasing trends of cannabis use in the Italian general population [14–16], based on the hypothesis that the true prevalence of current users could be approximately 20%, we estimated that, with a postulated survey response rate of 60%, a sample of 4700 patients (i.e., 2820 responders) would have guaranteed a margin of error of ± 1.5% of the 95% confidence interval (95% CI).

For data processing, surveys were downloaded from REDCap. Duplicates were identified, verified using age, gender, year of MS diagnosis, year of MS onset, MS form, and current DMT and then discarded from the database. Uncompleted surveys with missing data for the primary outcome or for variables used to identify duplicates were discarded.

The binomial method was used to estimate the 95% CI for the primary outcome. The Shapiro–Wilk test was used to test for normality. Data were presented as mean and standard difference (SD), median and interquartile range (IQR), or counts and percentages, as applicable. ANOVA, Kruskal–Wallis, Student’s t, Wilcoxon rank-sum, Chi-squared, or Fisher’s exact tests were used as applicable for comparisons. A *p* value < 0.05 was considered significant. Exploratory analyses were performed in the current users population, comparing patients who reported any medical use (i.e., current medical users) to those reporting only recreational use (i.e., current recreational users). A sensitivity analysis only in patients with RRMS was performed. We used STATA 16 (StataCorp. 2019. Stata Statistical Software: Release 16. College Station, TX: StataCorp LLC) for the analyses.

## Results

### Study population and characteristics

**Fig. 1:**
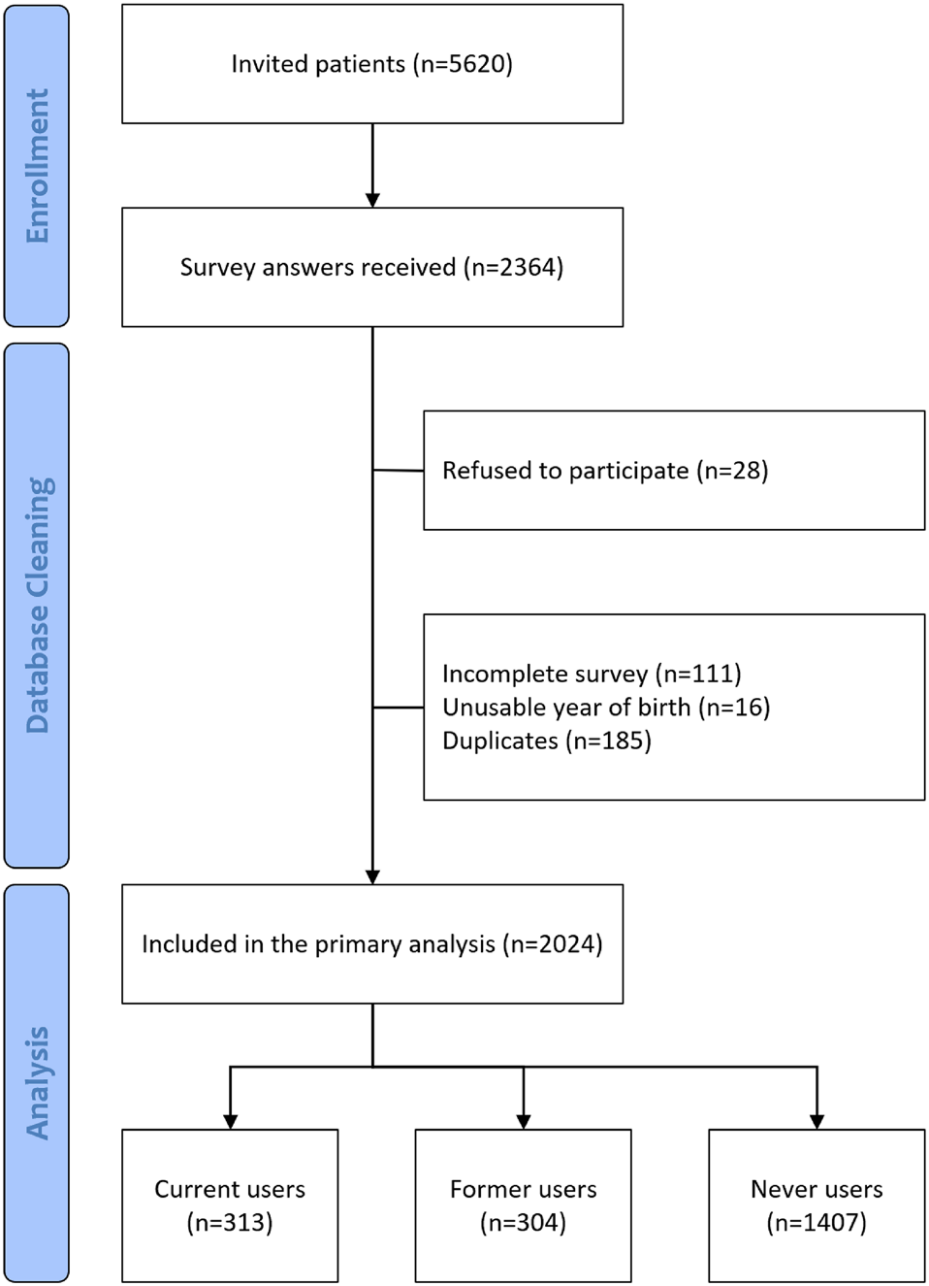
Study flow-diagram

We contacted 5620 patients and received 2364 answers. After database cleaning, a total of 2024 patients were included in the primary analysis (response rate 36.0%) (Fig. 1). The study population had a mean age of 45.2 years, constituted mainly of patients with RRMS (77.3%), followed by patients with SPMS (6.6%), PPMS (3.6%), and CIS (1.5%), while 223 (11.0%) patients could not define their disease phenotype. Participants were more frequently female (64.5%), Italian (97.3%) living in Lombardy (78.3%) (Supplementary Table S1). The mean MS duration from onset and from diagnosis resulted 14.0 and 12.2 years, respectively. Only 37.5% patients were able to report their last EDSS score, with a median of 2.0 (IQR 1.5–4.0) points, while median PDDS was 0 (IQR 0–3) points. Spasticity and pain were reported by 23.1% and 24.9% patients, with mean VAS score of 5.3 and 5.5, respectively. 90.7% patients were receiving a DMT (Supplementary Table S2). Concomitant neurological/psychiatric drugs or analgesics were assumed by 25.8% and 25.4% patients, respectively, while 6.5% participants received a cannabinoid prescription in the last 12 months. Complete study population characteristics are reported in Tables 1, 2, 3, 4, 5 and 6.

**Table 1:** Main sociodemographic and clinical characteristics

Variable	Total population (*n* = 2024)	Current user (*n* = 313)	Former user (*n* = 304)	Never user (*n* = 1407)	*p* value	Current medical user (*n* = 154)	Current recreational user (*n* = 159)	*p* value
Age, years					**0.0001**			0.1545
Mean (SD)	45.2 (11.4)	38.8 (10.1)	41.7 (9.6)	47.4 (11.4)		39.8 (10.6)	37.8 (9.5)	
Median (IQR)	46 (36–54)	37 (31–46)	42 (34–49)	48 (39–56)		39.5 (31–47)	36 (31–45)	
Gender, *n* (%)					< **0.0001**			0.308
Female	1306 (64.5)	148 (47.3)	165 (54.3)	993 (70.6)		78 (50.7)	70 (44.0)	
Non binary	2 (0.1)	1 (0.3)	0 (0.0)	1 (0.1)		0 (0.0)	1 (0.6)	
Prefer not to answer	2 (0.1)	1 (0.3)	0 (0.0)	1 (0.1)		0 (0.0)	1 (0.6)	
MS duration from onset, years					**0.0001**			0.0943
Mean (SD)	14.0 (9.5)	11.0 (7.9)	13.0 (8.7)	14.9 (9.8)		11.5 (7.3)	10.6 (8.4)	
Median (IQR)	12 (7–20.5)	9 (5–15)	11 (6–19)	13 (7–22)		10 (6–16)	8 (4–14)	
MS duration from diagnosis, years					**0.0001**			0.2018
Mean (SD)	12.2 (8.8)	9.5 (7.5)	11.2 (8.0)	13.0 (9.1)		9.9 (7.2)	9.1 (7.7)	
Median (IQR)	10 (5–18)	7 (4–13)	9 (5–15)	11 (6–19)		8 (4–14)	7 (3–12)	
MS form					0.497			0.234
CIS	31 (1.5)	6 (1.9)	7 (2.3)	18 (1.3)		4 (2.6)	2 (1.3)	
RRMS	1564 (77.3)	241 (77.0)	246 (80.9)	1077 (76.6)		115 (74.7)	126 (79.3)	
SPMS	133 (6.6)	16 (5.1)	19 (6.3)	98 (7.0)		11 (7.1)	5 (3.1)	
PPMS	73 (3.6)	12 (3.8)	7 (2.3)	54 (3.8)		8 (5.2)	4 (2.5)	
Do not know	223 (11.0)	38 (12.1)	25 (8.2)	160 (11.4)		16 (10.4)	22 (13.8)	
Patients aware of their EDSS score, *n* (%)	759 (37.5)	103 (32.9)	121 (39.8)	535 (38.0)	0.160	63 (40.9)	40 (25.2)	**0.004**
EDSS, points, median (IQR)	2 (1.5–4)	2.5 (1.5–4.5)	2 (1–4)	2 (1.5–4)	0.1178	3 (2–5.5)	2 (1.0–3.5)	**0.0025**
PDDS, points, median (IQR)	0 (0–3)	0 (0–2)	0 (0–2)	0 (0–3)	0.1724	2 (0–3)	0 (0–1)	< **0.0001**
PDDS, *n* (%)								
From no to moderate disability, n (%)	1512 (74.7)	237 (75.7)	242 (79.6)	1033 (73.4)	0.072	99 (64.3)	138 (86.8)	< **0.0001**
0–no disability	1032 (51.0)	158 (50.5)	169 (55.6)	705 (50.1)		48 (31.2)	110 (69.2)	
1–mild disability	280 (13.8)	40 (12.8)	42 (13.89)	198 (14.1)		23 (14.9)	17 (10.7)	
2–moderate disability	200 (9.9)	39 (12.5)	31 (10.2)	130 (9.2)		28 (18.2)	11 (6.9)	
3–gait disability	250 (12.4)	37 (11.8)	30 (9.9)	183 (13.0)		27 (17.5)	10 (6.3)	
4–early cane	97 (4.8)	14 (4.5)	11 (3.6)	72 (5.1)		11 (7.1)	3 (1.9)	
5–late cane	32 (1.6)	2 (0.6)	2 (0.7)	28 (2.0)		2 (1.3)	0 (0.0)	
6–bilateral support	67 (3.3)	15 (3.89)	10 (3.3)	45 (3.2)		8 (5.2)	4 (2.5)	
7–wheelchair/scooter	63 (3.1)	10 (3.2)	9 (3.0)	44 (3.1)		6 (3.9)	4 (2.5)	
8–bedridden	3 (0.2)	1 (0.3)	0 (0.0)	2 (0.1)		1 (0.7)	0 (0.0)	
Spasticity, yes, *n* (%)	467 (23.1)	84 (26.8)	62 (20.4)	321 (22.8)	0.151	61 (39.6)	23 (14.5)	< **0.0001**
Spasticity, VAS					0.5216			0.3643
Mean (SD)	5.3 (2.4)	5.0 (2.7)	5.2 (2.2)	5.3 (2.4)		5.2 (2.7)	4.6 (2.7)	
Median (IQR)	5 (3–7)	5 (3.7)	6 (3–7)	5 (4–7)		5 (3–7)	5 (2–6)	
Pain, yes, *n* (%)	504 (24.9)	78 (24.9)	67 (22.0)	359 (25.5)	0.446	64 (41.6)	14 (8.8)	< **0.0001**
Pain, VAS					0.2134			0.1717
Mean (SD)	5.5 (2.0)	5.7 (1.9)	5.1 (2.0)	5.5 (2.0)		5.9 (2.0)	5.1 (1.6)	
Median (IQR)	5 (4–7)	6 (5–7)	5 (4–7)	5 (5–7)		6 (4.5–7)	5 (5–7)	
Spasm frequency, *n* (%)					0.357			< **0.0001**
No	1463 (72.3)	211 (67.4)	227 (74.7)	1025 (72.9)		81 (52.6)	130 (81.0)	
Mild spasms induced by stimulation	242 (12.0)	46 (14.7)	31 (10.2)	165 (11.7)		29 (18.8)	17 (10.7)	
Spasms occurring < 1 times per h	231 (11.4)	40 (12.8)	32 (10.5)	159 (11.3)		31 (20.1)	9 (5.7)	
Spasms occurring > 1 times per h	65 (3.2)	11 (3.5)	13 (4.3)	41 (2.9)		10 (6.5)	1 (0.6)	
Spasms occurring > 10 times per h	23 (1.1)	5 (1.6)	1 (0.3)	17 (1.2)		3 (2.0)	2 (1.3)	
Bowel or bladder impairment, *n* (%)					0.115			< **0.0001**
No	987 (48.8)	157 (50.2)	154 (50.7)	676 (48.1)		60 (39.0)	97 (61.0)	
Mild hesitation, urgency, or retention	566 (28.0)	84 (26.8)	97 (31.9)	385 (27.4)		47 (30.5)	37 (23.3)	
Moderate hesitation, urgency, or retention or stypsis, and/or rare urinary incontinence	357 (17.6)	54 (17.3)	43 (14.1)	260 (18.5)		36 (23.4)	18 (11.3)	
Frequent urinary incontinence; necessity of intermittent auto-catheterization; necessity of manual aid for bowel evacuation	59 (2.9)	14 (4.5)	5 (1.64)	40 (2.8)		10 (6.5)	4 (2.5)	
Almost constant catheterization	13 (0.6)	1 (0.3)	2 (0.6)	10 (0.7)		0 (0.0)	1 (0.6)	
Loss of bladder or bowel function; permanent catheterization	16 (0.8)	3 (1.0)	1 (0.3)	12 (0.9)		1 (0.7)	2 (1.3)	
Loss of bladder and bowel function	26 (1.3)	0 (0.0)	2 (0.6)	24 (1.7)		0 (0.0)	0 (0.0)	
MSQoL-29, mean (SD)								
Physical functioning	75.2 (32.7)	78.2 (30.5)	79.6 (30.6)	73.6 (33.5)	**0.0075**	68.7 (32.7)	88.0 (24.5)	< **0.0001**
Pain	77.6 (24.2)	77.6 (23.8)	8.9 (21.6)	77.0 (24.7)	0.1385	67.2 (25.3)	88.2 (16.5)	< **0.0001**
Emotional wellbeing	64.9 (19.2)	64.9 (18.3)	65.7 (18.7)	64.7 (19.6)	0.6788	61.7 (18.7)	68.2 (17.3)	**0.0036**
Energy	49.2 (21.5)	48.3 (19.9)	49.0 (19.9)	49.5 (22.1)	0.6687	43.3 (19.4)	53.4 (19.2)	< **0.0001**
Cognitive functioning	68.9 (22.6)	66.8 (23.5)	70.0 (21.8)	69.1 (22.6)	0.3020	62.8 (23.6)	71.0 (22.6)	**0.0017**
Health stress	72.8 (27.9)	74.3 (24.78)	74.1 (23.2)	72.1 (25.2)	0.3111	67.6 (26.0)	81.1 (21.4)	< **0.0001**
Sexual functioning	71.7 (33.0)	74.1 (31.8)	69.8 (3.9)	71.6 (33.1)	0.4035	70.3 (33.3)	78.0 (29.7)	**0.0201**
Change in health	47.7 (23.0)	51.6 (24.4)	49.5 (23.9)	64.4 (22.4)	**0.0018**	48.0 (26.4)	55.2 (21.7)	**0.0142**
Social functioning	63.8 (27.5)	62.6 (27.7)	65.8 (26.0)	63.6 (27.7)	0.3936	54.6 (27.7)	70.8 (25.2)	< **0.0001**
Health perception	45.9 (31.0)	46.9 (31.6)	46.8 (30.9)	45.4 (30.8)	0.7693	37.5 (29.9)	65.3 (30.5)	< **0.0001**
Overall QoL	67.3 (19.4)	67.0 (18.5)	28.9 (19.2)	67.0 (19.6)	0.2745	61.1 (18.9)	73.0 (16.1)	< **0.0001**
PCS	62.4 (19.7)	64.2 (19.1)	63.9 (18.7)	61.6 (20.0)	0.0754	57.5 (19.1)	71.0 (16.6)	< **0.0001**
MCS	64.9 (18.5)	64..5 (18.0)	66.2 (16.9)	64.7 (18.9)	0.5264	58.4 (18.3)	70.7 (15.5)	< **0.0001**
HADS								
Anxiety, mean (SD)	6.1 (4.2)	5.9 (4.0)	6.1 (4.1)	6.1 (4.3)	0.9118	6.5 (4.1)	5.3 (3.8)	**0.0133**
Depression, mean (SD)	4.1 (3.6)	3.9 (3.3)	3.8 (3.3)	4.1 (3.7)	0.7095	4.2 (3.5)	3.7 (3.2)	0.1636
Anxiety > 7 points, *n* (%)	728 (36.0)	110 (35.1)	109 (35.9)	509 (36.2)	0.945	58 (37.7)	52 (32.7)	0.407
Depression > 7 points, *n* (%)	367 (18.1)	54 (17.3)	51 (16.8)	262 (18.6)	0.708	29 (18.8)	25 (15.7)	0.550

Significant* p* values are in bold

*CIS* clinically isolated syndrome, *EDSS* expanded disability status scale, *HADS* hospital anxiety and depression scale, *IQR* interquartile range, *MCS* mental composite score, *MS* multiple sclerosis, *MSQoL-29* 29-questions Multiple Sclerosis Quality of Life, *PCS* physical composite score, *PDDS* patient determined disease steps, *PPMS* primary progressive MS, *QoL* quality of life, *RRMS* relapsing remitting MS, *SD* standard deviation, *SPMS* secondary progressive MS, *VAS* visual analogue scale

**Table 2:** Unprescribed cannabis consumption motivation

Variable	Total population (*n* = 2024)	Current user (*n* = 313)	Former user (*n* = 304)	Never user (*n* = 1407)	*p* value	Current medical user (*n* = 154)	Current recreational user (*n* = 159)	*p* value
Disclosure of cannabis use to the physician, yes, *n* (%)	–	114 (36.4)	–	–	–	72 (46.8)	42 (26.4)	< **0.001**
Cannabis current use motivation, *n* (%)					–			–
Recreational	–	159 (50.6)	–	–		–	–	
Medical	–	54 (17.3)	–	–		–	–	
Both	–	100 (32.1)	–	–		–	–	
Did the patient used cannabis before the last 12 months (former user), yes, *n* (%)	591 (29.2)	287 (91.7)	304 (100)	–	< **0.001**	136 (88.3)	151 (95.0)	**0.040**
Cannabis former use motivation, *n* (%)					< **0.001**			< **0.001**
Recreational	449 (76.0)	185 (64.5)	264 (86.8)	–		40 (29.4)	145 (96.0)	
Medical	43 (7.3)	30 (10.5)	13 (4.28)	–		29 (21.3)	1 (0.7)	
Both	99 (16.8)	72 (25.1)	27 (8.9)	–		67 (49.3)	5 (3.3)	
Who advised the patient to use cannabis for medical use, *n* (%)^a^								
Personal idea	–	97 (63.0)	17 (42.5)	–	**0.030**	97 (63.0)	4 (66.7)	1.000
Friends	–	39 (25.3)	12 (30.0)	–	0.550	39 (25.3)	1 (16.7)	1.000
Family	–	14 (9.1)	7 (17.5)	–	0.152	14 (9.1)	1 (16.7)	0.452
Internet	–	48 (31.2)	6 (15.0)	–	**0.048**	48 (31.2)	0 (0.0)	0.180
Patients’ groups	–	33 (21.4)	10 (25.0)	–	0.670	33 (21.4)	0 (0.0)	0.347
Physician	–	22 (14.3)	8 (20.0)	–	0.461	22 (14.3)	1 (16.7)	1.000
Other healthcare workers	–	7 (4.6)	1 (2.5)	–	1.000	7 (4.6)	1 (16.7)	0.269
Other	–	21 (13.6)	7 (17.5)	–	0.613	21 (13.6)	1 (16.7)	0.595
Cannabis use interruption motivation (multiple choices possible), *n* (%)					–			–
Lack of effect	–	–	18 (5.9)	–		–	–	
Adverse effects	–	–	50 (16.5)	–		–	–	
Fear of others judgement	–	–	20 (6.6)	–		–	–	
Financial problems	–	–	11 (3.6)	–		–	–	
Legal problems	–	–	20 (6.6)	–		–	–	
Other	–	–	222 (74.3)	–		–	–	
Patient would use cannabis if it was legal, *n* (%)	–	–	–	576 (41.0)	–	–	–	–
Reason of cannabis use if it was legal, *n* (%)					–			–
Recreational	–	–	–	37 (6.4)		–	–	
Medical	–	–	–	417 (72.4)		–	–	
Both	–	–	–	122 (21.2)		–	–	

Significant* p* values are in bold

^a^Analysis performed only on those who reported medical or both medical and recreational use among current and former users

**Table 3:** Other sociodemographic variables

Variable	Total population (*n* = 2024)	Current user (*n* = 313)	Former user (*n* = 304)	Never user (*n* = 1407)	*p* value	Current medical user (*n* = 154)	Current recreational user (*n* = 159)	*p* value
Instruction level, *n* (%)					0.113			**0.015**
None	2 (0.1)	0 (0.0)	0 (0.0)	2 (0.1)		0 (0.0)	0 (0.0)	
Elementary school	14 (0.7)	0 (0.0)	1 (0.3)	13 (0.9)		0 (0.0)	0 (0.0)	
Secondary school	244 (12.1)	44 (14.1)	31 (10.2)	169 (12.0)		19 (12.3)	25 (15.7)	
High school diploma	951 (47.0)	145 (46.3)	135 (44.4)	671 (47.7)		85 (55.2)	60 (37.7)	
University	604 (29.8)	81 (25.9)	105 (34.5)	418 (29.7)		35 (22.7)	46 (28.9)	
Master/PhD/Postgraduate specializations	209 (10.3)	43 (13.7)	32 (10.5)	134 (9.5)		15 (9.7)	28 (17.6)	
Work, *n* (%)					< **0.001**			**0.005**
Full time	1208 (59.7)	196 (62.6)	207 (68.1)	805 (57.2)		82 (53.3)	114 (71.7)	
Part time	361 (17.8)	57 (18.2)	56 (18.4)	248 (17.6)		34 (22.1)	23 (14.5)	
Unemployed	277 (13.7)	50 (16.0)	28 (9.2)	199 (14.1)		30 (19.5)	20 (12.6)	
Retirement	178 (8.8)	10 (3.2)	13 (4.3)	155 (11.0)		8 (5.2)	2 (1.3)	
Annual income, *n* (%)^a^					0.104			**0.002**
0–10,000 €	270 (15.3)	50 (17.9)	33 (12.0)	187 (15.5)		30 (22.6)	20 (13.6)	
10,000–15,000 €	276 (15.7)	49 (17.5)	38 (13.8)	189 (15.7)		23 (17.3)	26 (17.7)	
15,000–26,000 €	589 (33.4)	100 (35.7)	105 (38.0)	384 (31.8)		56 (42.1)	44 (29.9)	
26,000–55,000 €	466 (26.4)	64 (22.9)	79 (28.6)	323 (26.8)		22 (16.5)	42 (28.6)	
55,000–75,000 €	98 (5.6)	10 (3.6)	14 (5.1)	74 (6.1)		2 (1.5)	8 (5.4)	
75,000–120,000 €	53 (3.0)	4 (1.4)	7 (2.5)	42 (3.5)		0 (0.0)	4 (2.7)	
More than 120,000 €	11 (0.6)	3 (1.1)	0 (0.0)	8 (0.7)		0 (0.0)	3 (2.0)	
Prefer not to disclose	261	33	28	200		21	12	
Number of inhabitants of the municipality of residence, *n* (%)^a^					0.261			0.526
Less than 2000	90 (4.7)	13 (4.6)	14 (4.7)	63 (4.7)		8 (5.9)	5 (3.3)	
2000–10,000	503 (26.3)	65 (22.7)	76 (25.7)	362 (27.1)		37 (25.0)	31 (20.7)	
10,000–50,000	629 (32.8)	85 (29.7)	93 (31.4)	451 (33.8)		42 (30.9)	43 (28.7)	
50,000–250,000	288 (15.0)	52 (18.2)	41 (13.9)	195 (14.6)		23 (16.9)	29 (19.3)	
More than 250,000	402 (21.2)	71 (24.8)	72 (24.3)	263 (19.7)		29 (21.3)	42 (28.0)	
Do not know	108	27	8	73		18	9	
Marital status, *n* (%)					< **0.001**			1.000
Free	738 (36.5)	164 (52.4)	141 (46.3)	433 (30.8)		81 (52.6)	83 (52.2)	
Married	1122 (55.4)	127 (40.6)	138 (45.3)	857 (60.9)		62 (40.3)	65 (40.9)	
Separated/divorced	164 (8.1)	22 (7.0)	25 (8.2)	117 (8.3)		11 (7.1)	11 (6.9)	
Housing situation, *n* (%)					< **0.001**			0.773
Living with the partner	648 (32.0)	104 (33.23)	92 (30.3)	452 (32.1)		53 (34.4)	51 (32.1)	
Living with partner and children	721 (35.6)	72 (23.32)	105 (34.5)	543 (38.6)		33 (21.4)	40 (25.2)	
Living with children	96 (4.7)	10 (3.2)	20 (6.6)	66 (4.7)		6 (3.9)	4 (2.5)	
Living with parents or family of origin	244 (12.1)	62 (19.8)	35 (11.5)	147 (10.5)		33 (21.4)	29 (18.2)	
Living alone	315 (15.7)	64 (20.5)	52 (17.1)	199 (14.1)		29 (18.8)	35 (22.0)	

Significant* p* values are in bold

^a^Patients who preferred not to disclose the data or were not aware/did not know were excluded from the statistical comparison

**Table 4:** Concomitant medications and other substances

Variable	Total population (*n* = 2024)	Current user (*n* = 313)	Former user (*n* = 304)	Never user (*n* = 1407)	*p* value	Current medical user (*n* = 154)	Current recreational user (*n* = 159)	*p* value
DMT lines, *n* (%)^a^					0.185			0.146
First line	998 (49.3)	141 (45.1)	138 (49.3)	719 (51.1)		60 (39.0)	81 (50.9)	
Second line	806 (39.8)	141 (45.1)	806 (39.8)	532 (37.8)		75 (48.7)	66 (41.5)	
Other	32 (1.6)	6 (1.9)	4 (1.3)	22 (1.6)		4 (2.6)	2 (1.3)	
None	188 (9.3)	25 (8.0)	29 (9.5)	134 (9.5)		15 (9.7)	10 (6.3)	
Concomitant neurological/psychiatric drugs, *n* (%)	523 (25.8)	79 (25.2)	69 (22.7)	375 (26.7)	0.384	53 (34.4)	26 (16.4)	< **0.001**
Gabapentin or pregabalin	178 (8.8)	24 (7.7)	16 (5.3)	138 (9.8)		18 (11.7)	6 (3.8)	
Anti-convulsants	38 (1.9)	4 (1.3)	3 (1.0)	31 (2.2)		3 (2.0)	1 (0.6)	
Anti-depressants	166 (8.2)	25 (8.0)	33 (10.9)	108 (7.7)		14 (9.1)	11 (6.9)	
Benzodiazepines	159 (7.9)	23 (7.4)	19 (6.3)	117 (8.3)		20 (13.0)	6 (1.9)	
Antipsychotics	14 (0.7)	1 (0.3)	2 (0.7)	11 (0.8)		1 (0.7)	0 (0.0)	
Lithium or valproate	9 (0.4)	2 (0.6)	0 (0.0)	7 (0.5)		1 (0.7)	1 (0.6)	
Anti-spastics	127 (6.3)	29 (9.3)	15 (4.9)	83 (5.9)		21 (13.6)	8 (5.0)	
Concomitant analgesic drugs, *n* (%)	515 (25.4)	69 (22.4)	67 (22.0)	379 (26.9)	0.067	48 (31.2)	21 (13.2)	< **0.001**
Paracetamol	304 (15.0)	36 (11.5)	39 (12.8)	222 (16.3)		23 (14.9)	13 (8.2)	
NSAIDs	287 (14.2)	48 (15.3)	39 (12.8)	200 (14.2)		36 (23.4)	12 (7.6)	
Codeine or tramadol	21 (1.0)	4 (1.3)	0 (0.0)	17 (1.2)		2 (1.3)	2 (1.3)	
Strong opioids	19 (0.9)	1 (0.3)	1 (0.3)	17 (1.2)		0 (0.0)	1 (0.6)	
Prescription cannabinoids in the last 12 months, *n* (%)	131 (6.5)	45 (14.4)	12 (4.0)	74 (5.3)	< **0.001**	41 (26.6)	4 (2.5)	< **0.001**
Nabiximols	89 (4.4)	21 (6.7)	8 (2.6)	60 (4.3)	–	19 (12.3)	2 (1.3)	
Bedrocan, Bedrobinol, Bedica	11 (0.5)	6 (1.9)	0 (0.0)	5 (0.4)	–	5 (3.3)	1 (0.6)	
Bediol	5 (0.3)	3 (1.0)	1 (0.3)	1 (0.1)	–	2 (1.3)	1 (0.6)	
Bedrolite	4 (0.2)	4 (1.3)	0 (0.0)	0 (0.0)	–	3 (2.0)	1 (0.6)	
Dronabinol	2 (0.1)	1 (0.3)	0 (0.0)	1 (0.1)	–	0 (0.0)	1 (0.6)	
FM1	8 (0.4)	3 (1.0)	2 (0.7)	3 (0.2)	–	2 (1.3)	1 (0.6)	
FM2	3 (0.2)	2 (0.6)	0 (0.0)	1 (0.1)	–	1 (0.7)	1 (0.6)	
Others	30 (1.5)	23 (7.4)	2 (0.7)	5 (0.4)	–	19 (12.3)	4 (2.5)	
Alcohol use, *n* (%)					< **0.001**			**0.032**
No	776 (38.4)	64 (20.5)	73 (24.1)	639 (45.5)		41 (26.6)	23 (14.6)	
Daily	149 (7.4)	27 (8.7)	30 (9.9)	92 (6.5)		8 (5.2)	19 (2.0)	
2–3 times weekly	282 (14.0)	68 (21.8)	51 (16.8)	163 (11.6)		29 (18.8)	39 (24.7)	
1 time weekly	371 (18.4)	78 (25.0)	70 (23.1)	223 (15.8)		37 (24.0)	41 (26.0)	
1–2 times monthly	277 (13.7)	59 (18.9)	50 (16.5)	168 (12.0)		29 (18.8)	30 (19.0)	
Less than 1 time monthly	166 (8.2)	16 (5.1)	29 (9.6)	121 (8.6)		18 (6.5)	6 (3.8)	
Tobacco smoke, *n* (%)	565 (28.0)	189 (60.6)	116 (38.3)	260 (18.5)	< **0.001**	92 (59.7)	97 (61.4)	0.817
Other psychoactive substances use, *n* (%)^b^					< **0.001**			1.000
Yes	20 (1.0)	12 (4.0)	3 (1.0)	5 (0.4)		6 (4.0)	6 (3.9)	
Prefer not to disclose	22	8	0	14		3	5	
Other psychoactive substances used, *n* (%)								
Opioids	6 (30.0)	2 (16.7)	1 (33.3)	3 (60.0)	0.205	1 (16.7)	1 (16.7)	1.000
Cocaine	15 (75.0)	10 (83.3)	3 (100.0)	2 (40.0)	0.147	4 (66.7)	6 (100.0)	0.455
Stimulants	6 (30.0)	4 (33.3)	1 (33.3)	1 (20.0)	1.000	3 (50.0)	1 (16.7)	0.545
Ketamine	2 (10.0)	2 (16.7)	0 (0.0)	0 (0.0)	1.000	1 (16.7)	1 (16.7)	1.000
Hallucinogens	4 (20.0)	3 (25.0)	1 (33.3)	0 (0.0)	0.569	2 (33.3)	1 (16.7)	1.000
Others	1 (5.0)	1 (8.3)	0 (0.0)	0 (0.0)	1.000	0 (0.0)	1 (16.7)	1.000

Significant* p* values are in bold

*DMT* disease modifying treatment, *NSAIDs* non-steroidal anti-inflammatory drugs

^a^First line DMTs included glatiramoids, interferons, dimethyl fumarate, and teriflunomide; second line DMTs included fingolimod, siponimod, ozanimod, ponesimod, cladribine, natalizumab, ocrelizumab, ofatumumab, rituximab, and alemtuzumab; other included other immunosuppressants and investigational drugs in clinical trial

^b^Patients who preferred not to disclose the data were excluded from the statistical comparison

**Table 5:** Cannabis use characteristics

Variable	Total population (*n* = 2024)	Current user (*n* = 313)	Former user (*n* = 304)	Never user (*n* = 1407)	*p* value	Current medical user (*n* = 154)	Current recreational user (*n* = 159)	*p* value
Main source of cannabis supply, *n* (%)					< **0.001**			0.449
Friends or family	–	116 (56.9)	156 (64.5)	–		48 (53.3)	68 (59.7)	
Street market	–	64 (31.4)	82 (33.9)	–		28 (31.1)	36 (31.6)	
Dark web	–	8 (3.9)	1 (0.4)	–		4 (4.4)	4 (3.5)	
Own cultivation	–	16 (7.8)	3 (1.2)	–		10 (11.1)	6 (5.3)	
Prefer not to disclose^a^	–	109	59	–		64	45	
Issues related to cannabis use, *n* (%)					< **0.001**			**0.003**
Legal issues	–	10 (3.5)	7 (2.5)	–		5 (3.7)	5 (3.4)	
Supply issues	–	67 (23.6)	22 (7.9)	–		43 (31.4)	24 (16.3)	
Both legal and supply issues	–	16 (5.6)	6 (2.14)	–		11 (8.0)	5 (3.4)	
None	–	191 (67.3)	245 (87.5)	–		78 (56.9)	113 (76.9)	
Prefer not to disclose^a^	–	29	24	–		17	12	
Weekly cannabis cost estimate, *n* (%)					< **0.001**			**0.005**
0–5 €	–	87 (36.4)	123 (63.7)	–		32 (27.6)	55 (44.7)	
5–10 €	–	43 (18.0)	36 (18.7)	–		16 (13.8)	27 (22.0)	
10–25 €	–	54 (22.6)	19 (9.8)	–		32 (27.6)	22 (17.9)	
25–50 €	–	34 (14.2)	11 (5.7)	–		21 (18.1)	13 (10.6)	
50–100 €	–	15 (6.3)	2 (1.0)	–		11 (9.5)	4 (3.3)	
More than 100 €	–	6 (2.5)	2 (1.0)	–		4 (3.5)	2 (1.6)	
Do not know^a^	–	51	94	–		24	27	
Prefer not to disclose^a^	–	22	16	–		14	8	
Principal way of cannabis assumption, *n* (%)					**0.001**			< **0.001**
Smoked (cigarette)	–	260 (83.3)	281 (92.4)	–		114 (74.0)	146 (92.4)	
Smoked (pipe)	–	2 (0.6)	5 (1.6)	–		2 (1.3)	0 (0.0)	
Vaped (electronic cigarette)	–	3 (1.0)	1 (0.3)	–		1 (0.7)	2 (1.3)	
Vaped (vaporization)	–	12 (3.9)	1 (0.3)	–		9 (5.8)	3 (1.9)	
Infuse	–	11 (3.5)	5 (1.6)	–		9 (5.8)	2 (1.3)	
Cooked (cookies, cakes)	–	2 (0.6)	4 (1.3)	–		2 (1.3)	0 (0.0)	
Ingestion as oil	–	19 (6.1)	4 (1.3)	–		15 (9.7)	4 (2.5)	
Other	–	3 (1.0)	3 (1.0)	–		2 (1.3)	1 (0.6)	
Frequency of cannabis administration, *n* (%)					< **0.001**			< **0.001**
Daily	–	126 (40.5)	29 (9.5)	–		74 (48.1)	52 (33.1)	
5–6 days per week	–	13 (4.2)	6 (2.0)	–		11 (7.1)	2 (1.3)	
3–4 days per week	–	44 (14.2)	24 (7.9)	–		20 (13.0)	24 (15.3)	
1–2 times monthly	–	45 (4.5)	77 (25.3)	–		28 (18.2)	17 (10.8)	
Less than 1 time monthly	–	83 (26.7)	168 (55.3)	–		21 (13.6)	62 (39.5)	
Type of assumed cannabis, *n* (%)					**0.006**			**0.009**
High THC (e.g., hashish, marijuana)	–	227 (77.0)	189 (86.7)	–		105 (70.5)	122 (83.6)	
Only CBD (e.g., cannabis light)	–	68 (23.1)	29 (13.3)	–		44 (29.5)	24 (16.4)	
Not aware^a^	–	18	86	–		5	13	
Amount of consumed cannabis variation, *n* (%)					< **0.001**			< **0.001**
Increased during time	–	24 (7.7)	1 (0.3)	–		15 (9.8)	9 (5.7)	
Reduced during time	–	85 (27.3)	212 (70.0)	–		21 (13.7)	64 (40.5)	
Varying depending on the period	–	82 (26.4)	29 (9.6)	–		52 (34.0)	30 (19.0)	
Unvaried	–	120 (38.6)	61 (20.1)	–		65 (42.5)	55 (34.8)	
Variability of cannabis effects, *n* (%)					0.358			0.089
Yes	–	71 (29.6)	48 (34.5)	–		42 (35.0)	29 (24.2)	
No	–	169 (70.4)	91 (65.5)	–		78 (56.0)	91 (75.8)	
Not know/not remember^a^	–	73	164	–		34	39	
Type of variability, *n* (%)					0.606			0.138
Increased effects	–	25 (35.2)	16 (33.3)	–		12 (28.6)	13 (44.8)	
Reduced effects	–	15 (21.1)	7 (14.6)	–		12 (28.6)	3 (10.3)	
Unexpected effects	–	31 (43.7)	25 (52.1)	–		18 (42.9)	13 (44.8)	
Cannabis-related adverse effects, *n* (%)		203 (64.9)	186 (61.2)		0.360	91 (59.1)	112 (70.4)	**0.044**
Dry mouth	–	130 (64.0)	91 (48.9)	–	**0.003**	62 (68.1)	68 (60.7)	0.305
Mucosal irritation	–	11 (5.4)	7 (3.8)	–	0.478	3 (3.3)	8 (7.1)	0.351
Weakness	–	31 (15.3)	31 (16.7)	–	0.782	13 (14.3)	18 (16.1)	0.845
Dizziness	–	42 (20.7)	78 (41.9)	–	< **0.001**	21 (23.1)	21 (18.8)	0.489
Tachycardia	–	72 (35.5)	63 (33.9)	–	0.750	27 (29.7)	45 (40.2)	0.141
Movement disorders	–	25 (12.3)	20 (10.8)	–	0.639	11 (12.1)	14 (12.5)	1.000
Anxiety	–	42 (20.7)	53 (28.5)	–	0.078	18 (19.8)	24 (21.4)	0.862
Panic	–	14 (6.9)	18 (9.7)	–	0.359	5 (5.5)	9 (8.4)	0.583
Hallucinations	–	6 (3.0)	15 (8.1)	–	**0.041**	1 (1.1)	5 (4.5)	0.227
Gastrointestinal disturbances	–	4 (2.0)	4 (2.2)	–	1.000	2 (2.2)	2 (1.8)	1.000
Sedation	–	15 (7.4)	29 (12.4)	–	0.124	10 (11.0)	5 (4.5)	0.105
Insomnia	–	10 (4.9)	7 (3.8)	–	0.627	6 (6.6)	4 (3.6)	0.348
Memory disturbances	–	43 (21.2)	20 (10.8)	–	**0.006**	23 (25.3)	20 (17.9)	0.228
Other	–	19 (9.4)	18 (9.7)	–	1.000	8 (8.8)	11 (9.8)	1.000
Cannabinoids consume modifications due to COVID-19 pandemic or lockdown, *n* (%)					–			0.123
Increased	–	46 (14.8)	–	–		25 (16.2)	21 (13.4)	
Decreased	–	87 (28.0)	–	–		35 (22.7)	52 (33.1)	
Unvaried	–	178 (57.2)	–	–		94 (61.0)	84 (53.5)	
Reason for increase (COVID-19 or lockdown), *n* (%)					–			**0.014**
Coping difficulty	–	10 (21.7)	–	–		8 (32.0)	2 (9.5)	
Symptoms worsening	–	3 (6.5)	–	–		3 (12.0)	0 (0.0)	
Recreational	–	28 (60.9)	–	–		10 (40.0)	18 (85.7)	
Do not know	–	1 (2.2)	–	–		1 (4.0)	0 (0.0)	
Other reasons	–	4 (8.7)	–	–		3 (12.0)	1 (4.8)	
Reason for decrease (COVID-19 or lockdown), *n* (%)					–			0.061
Supply difficulty	–	46 (52.9)	–	–		22 (62.9)	24 (46.2)	
Less need for recreational use	–	21 (24.1)	–	–		4 (11.4)	17 (32.7)	
Do not know	–	4 (4.6)	–	–		3 (8.6)	1 (1.9)	
Other reasons	–	16 (18.4)	–	–		6 (17.1)	10 (19.2)	

Significant* p* values are in bold

*CBD* cannabidiol, *THC* tetrahydrocannabinol

^a^Patients who preferred not to disclose the data or were not aware/did not know were excluded from the statistical comparison

**Table 6:** Cannabis effects on symptoms and medications modification

Variable	Total population (*n* = 2024)	Current user (*n* = 313)	Former user (*n* = 304)	Never user (*n* = 1407)	*p* value	Current medical user (*n* = 154)	Current recreational user (*n* = 159)	*p* value
Any MS-related symptoms improved by cannabis assumption, *n* (%)					< **0.001**			< **0.001**
Yes	–	177 (88.5)	46 (37.4)	–		128 (97.7)	49 (71.0)	
Not know^a^	–	111	181	–		22	89	
Symptoms improved by cannabis assumption, *n* (%)								
Pain	–	91 (51.4)	22 (47.8)	–	0.665	75 (58.6)	16 (32.7)	**0.002**
Spams or tremor	–	96 (54.2)	22 (47.8)	–	0.438	76 (59.4)	20 (40.8)	**0.030**
Sleep disturbances	–	117 (66.1)	28 (60.9)	–	0.507	91 (71.1)	26 (53.1)	**0.033**
Bowel or bladder disturbances	–	27 (15.3)	5 (10.9)	–	0.450	20 (15.6)	7 (14.3)	1.000
Appetite	–	44 (24.9)	5 (10.9)	–	**0.041**	33 (25.8)	11 (22.5)	0.702
Anxiety	–	90 (50.9)	17 (37.0)	–	0.093	67 (52.3)	23 (46.9)	0.615
Mood	–	73 (41.2)	13 (28.3)	–	0.107	55 (43.0)	18 (36.7)	0.498
Coping	–	31 (17.5)	4 (8.7)	–	0.143	22 (17.2)	9 (18.4)	0.828
Nausea, vomiting, and GI disturbances	–	13 (7.3)	1 (2.2)	–	0.198	12 (9.4)	1 (2.0)	0.116
Headache	–	43 (24.3)	11 (23.9)	–	0.957	31 (34.2)	12 (24.5)	1.000
Adverse effects of other drugs	–	14 (7.9)	1 (2.2)	–	0.166	14 (10.9)	0 (0.0)	**0.012**
MS improvement	–	61 (34.5)	10 (21.7)	–	0.099	50 (39.1)	11 (22.5)	0.051
Sensory symptoms	–	65 (36.7)	21 (45.7)	–	0.268	47 (36.7)	18 (36.7)	1.000
Others	–	29 (16.4)	2 (4.3)	–	**0.036**	22 (17.2)	7 (14.3)	0.821
Drugs for anxiety, sleep, pain, depression, or other conditions reduced due to cannabis assumption, *n* (%)					**0.003**			< **0.001**
Yes, dose reduction of some drugs	–	39 (28.1)	8 (15.4)	–		30 (30.6)	9 (22.0)	
Yes, discontinuation of some drugs	–	51 (36.7)	11 (21.2)	–		45 (45.9)	6 (14.6)	
No, unvaried drugs assumption	–	49 (35.3)	33 (63.5)	–		23 (23.5)	26 (63.4)	
No, never assumed these kinds of drugs^b^	–	118	133	–		37	81	
Not know^b^	–	54	119	–		18	36	

Significant* p* values are in bold

^a^Patients who did not know were excluded from the statistical comparison

^b^Patients who did not know or never assumed these kinds of drugs were excluded from the statistical comparison

## Prevalence of *cannabis* consumption, motivations, and sociodemographic variables

In the primary analysis, the proportion of current users (i.e., participants who used unprescribed cannabinoids in the last 12 months) was 15.5% (95% CI 13.9–17.1); 15.0% patients were former users while 69.5% never used cannabis in life. Among current users, only 36.4% patients disclosed to their physician their unprescribed cannabis use. Current users more frequently reported medical or both medical and recreational use (49.4%) compared to former users (13.2%) (*p* < 0.001). Among current users who also reported a former use, only 35.6% reported medical or both medical and recreational use, still being significantly higher than former users (*p* < 0.001). The proportion of disclosures was significantly higher among current medical users compared to current recreational users (*p* < 0.001). (Table 2).

The most frequently reported sources of advice for medical use in current users were personal idea (63.0%), internet (31.2%), friends (25.3%), and patients’ groups (21.4%), with current users more frequently reporting personal idea (*p* = 0.030) and internet (*p* = 0.048) compared to former users. 41.0% of never users reported they would use cannabis if it was legal, mainly for medical purpose. 8.3% of current users started using unprescribed cannabis in the last year. Of them, 57.7% reported a medical use and 11.4% both medical and recreational use (Table 2).

Current users were younger (*p* = 0.0001) and were more frequently male (*p* < 0.0001) (Table 1); they more frequently reported a free marital status and living alone or with parents or family of origin. Significant differences were observed for work (*p* < 0.001); current medical users less frequently reported university or higher instruction level (*p* = 0.015), full time work (*p* = 0.005), and had an inferior annual income (*p* = 0.002) compared to current recreational users (Table 3).

### MS clinical variables and *cannabis* use

Current users had a shorter mean MS duration from onset and from diagnosis (*p* = 0.0001), with non-significant differences between groups for other MS-related clinical variables (Table 1). MS duration from onset and diagnosis was non-significantly different between current medical and recreational users. Conversely, current medical users had higher EDSS (*p* = 0.0025), PDDS (p < 0.0001), spasticity and pain (*p* < 0.0001), spasm frequency (*p* < 0.0001), and bowel or bladder impairment (*p* < 0.0001) compared to current recreational users (Table 1).

Concomitant medications were comparable between groups, while prescription cannabinoids in the last 12 months were more frequently reported in current users (*p* < 0.001). Current medical users showed increased proportions of concomitant neurologic/psychiatric drugs (*p* < 0.001), concomitant analgesics (*p* < 0.001), and prescription cannabinoids in the last 12 months (*p* < 0.001) while no difference was observed for DMT categories (*p* = 0.146) (Table 4).

HADS and MSQoL-29 items were non-significantly different apart from superior physical functioning in current and former users (*p* = 0.0075) and superior change in health in never users (*p* = 0.0018). Differently, current medical users showed significantly reduced scores in all MSQoL-29 items compared to current recreational users (*p* ranging 0.0036 to < 0.0001); they also showed significantly higher anxiety scores (*p* = 0.0133), while depression score and the proportion of patients having anxiety and depression were non-significantly different (Table 1).

### Other drugs and substances use

Current users more frequently assumed alcohol or tobacco smoke (*p* < 0.001), as well as other psychoactive substances compared to former and never users (*p* < 0.001) (Table 4). Other substances use was limited to only 1.0% of the overall population. Current medical users showed significantly reduced alcohol consumption compared to current recreational users (*p* = 0.032), while no significant differences were observed for tobacco smoke and other psychoactive substances (Table 4).

### *Cannabis* use characteristics

The two most frequent supply sources were friends or family and the street market with significant differences between groups (*p* < 0.001), with current users more frequently reporting own cultivation (7.8%) and the dark web (3.9%). Current users more frequently reported issues related to cannabis use (*p* < 0.001), in particular supply issues (Table 5).

Current users generally had a significantly higher weekly cannabis cost estimate compared to former users (*p* < 0.001). The frequency of cannabis consumption resulted significantly different (*p* < 0.001), with 58.9% of current users reporting from 3 days per week to daily administrations, while 80.6% of former users reported from two to less than one time monthly administrations. The principal way of consumption was smoked as cigarettes. Vaped forms, infuses, and ingestion as oil were more represented in current users (*p* = 0.001), which also more frequently assumed CBD-only products (*p* < 0.001). Current users more frequently reported increased, unvaried, or varying depending on the period cannabis use over time (*p* < 0.001). About a third of patients in both groups reported some variability of cannabis effects, most frequently unexpected, followed by increased effects. About two thirds of cannabis users experienced cannabis-related adverse effects. Current users reported significantly more frequently dry mouth (*p* = 0.003) and memory disturbances (*p* = 0.006), while dizziness (*p* < 0.001) and hallucinations (*p* = 0.041) were more frequent in former users (Table 5).

Current medical users reported more issues related to cannabis use, especially supply issues, (*p* = 0.003) superior weekly cost estimate (*p* = 0.005), more frequent alternative ways to smoked cannabis (*p* < 0.001), increased frequency of cannabis consumption (*p* < 0.001), more frequent use of CBD-only products (*p* = 0.009), and a consumed cannabis amount generally stable or varying depending on the period (*p* < 0.001), as well as significantly less cannabis-related adverse effects (*p* = 0.044), while the difference was non-significant for the individual investigated adverse effects (Table 5).

COVID-19 impact on cannabis consumption is reported in Table 5.

### *Cannabis* effects on symptoms and medications modification

Current users more frequently reported an improvement of MS-related symptoms with cannabis assumption (*p* < 0.001). The difference was significant also when comparing current medical users to current recreational users (*p* < 0.001). Pain, spasms or tremor, sleep disturbances, anxiety, and sensory symptoms were the most frequently improved symptoms. Current medical users more frequently reported significant improvements in pain (*p* = 0.002), spams or tremor (*p* = 0.030), sleep disturbances (*p* = 0.033), and adverse effects of other drugs (*p* = 0.012). Current users more frequently reported a dose reduction or a discontinuation of drugs for anxiety, sleep, pain, or other conditions compared to former users (*p* = 0.003). This finding was replicated in current medical users when compared to current recreational users (*p* < 0.001) (Table 6).

### Sensitivity analysis in patients with RRMS

Since a large part of included patients had RRMS, we performed a sensitivity analysis in this subpopulation, which yielded the same result for the primary outcome (i.e., current users prevalence 15.4%; 95% CI 13.7–17.3). The sensitivity analysis substantially replicated the results of the primary analysis (Supplementary Tables S3 to S8). The only differences consisted in non-significant differences for MSQoL-29 sexual functioning and change in health scores and for HADS anxiety score when comparing current medical users to current recreational users (Supplementary Table S3), minimal significant and non-significant differences in who advised the patient to use cannabis for medical use (Supplementary Table S4), significant differences in concomitant analgesic drugs between current, former, and never users (Supplementary Table S6), non-significant differences in weekly cost estimates between current medical users and current recreational users, non-significant differences in hallucinations reporting and significant differences in sedation reporting between current and former users (Supplementary Table S7), and non-significant differences in improvements of appetite and other symptoms due to cannabis assumption between current and former users (Supplementary Table S8).

## Discussion

In an Italian MS population followed at hospital centers in Lombardy Region, we identified a prevalence of current cannabis users of 15.5%. This result is superior to available estimates in the Italian general population in 2017 and similar or slightly inferior to reports from Denmark and UK [10, 14, 16]. Also, the prevalence of cannabis use in the Italian general population during 2022 was 8.5% [20]. Altogether, these results indicate that cannabis use might be more frequent in the Italian MS population, although this evidence could be limited being it from an indirect comparison with general population estimates and not from a direct case–control study. Still, given that general population could also use unprescribed cannabis with medical intent besides recreational use, it is well possible that such difference in prevalence might be due to different behaviour and attitudes in MS patients as compared to the general population. This hypothesis should be investigated in the future. Studies conducted in the USA and Canada showed superior prevalence of current (ranging 30.0–66.4%) and ever users, possibly due to different legislations enforced in these countries, where some states have already legalized cannabis [11–13]. The legal deterrent was relevant in MS patients, since 41.0% of never users would use cannabis if it was legal, mainly with medical intent. This is in line with data from Denmark, while in UK and Canada percentages of patients supporting cannabis legalization exceeded 70% [10, 12, 14]. A previous Italian study on 2009 data showed about 12% of ever users (i.e., current and former users) in patients with MS, while this proportion was 30.5% in our study [17]. This observation may be due to increasing trends in cannabis consumption in the general population [15]. However, whether this is the main reason or other reasons (e.g., knowledge of possible efficacy on MS symptoms, other curative beliefs) may contribute to the increased cannabis use in MS should be further studied. The frequent reporting of personal idea and internet instead of medical advice as sources supporting medical use, along with the limited number of patients who received prescription cannabinoids compared to the larger number of patients with pain and spasticity, could also indicate a reduced propensity of clinicians to discuss or prescribe medical cannabis. On the other hand, a quite considerable part of current users and current medical users disclosed their cannabis use to the physician, similarly to a previous Italian study [17]. Moreover, 14.4% of current users received a prescription cannabinoid in the previous year, indicating a potential risk of additional cannabinoids exposure, which should be considered when prescribing medical cannabis. Whether these prescription cannabinoids were interrupted for any reasons and patients initiated or continued to use unprescribed cannabis for medical use should be investigated in future studies. Nevertheless, it is noteworthy that while a considerable proportion of current and current medical users reported a clinical benefit of unprescribed cannabis use, only a minor part of them received a prescription cannabinoid.

Current users were more frequently male and younger, confirming previous findings [12–14]. Also, they more frequently had a free marital status and were living alone or with the family of origin [13, 14]. Younger age might explain these findings and the observed association of current users to a shorter MS duration. Current medical users reported superior disability, more frequent pain, spasticity and spasm frequency, and reduced quality of life. Similarly, in previous studies cannabis was used mainly by patients with superior disability [10, 12]. However, we did not observe a significant association between progressive forms and cannabis use as in Denmark and the USA, possibly due to the limited number of patients with PPMS and SPMS in our cohort [13, 14]. Given the results of our sensitivity analysis on RRMS patients only, it is also possible that in our cohort cannabis use might be somehow associated with disability severity regardless its origin (e.g., disability accumulation after relapses, progression-related disability) rather than with progression itself. These considerations are supported also by the significantly reduced quality of life by means of MSQoL-29 scores in current medical users. Consistently with previous reports [10], our patients declared clinical benefit from cannabis use especially for pain, spasms or tremor, sleep disturbances, and anxiety. This is not surprising given the indications for medicinal cannabis, which may also explain the reported benefit on sleep disturbances.

Current medical users had a significantly higher HADS anxiety score compared to current recreational users and both reported anxiety improvement with cannabis use, while the frequency of patients with possible anxiety was comparable. This may be due to the potential cannabis anxiolytic effect and the common presence of anxiety regardless the severity of MS [21]. The paradoxical observation that patients with anxiety report benefits from cannabis use and that cannabis use was associated with increased anxiety might be due to a dose-dependent phenomenon, where low doses of cannabinoids might be effective on anxiety while high-THC products may elicit anxiety. This topic is further discussed in a dedicated publication [22]. Cannabis use was associated to a reduction/discontinuation of medications for anxiety, sleep, pain, depression or other conditions in current medical users. Albeit needing confirmation in longitudinal studies, this observation is relevant since concomitant medications reduction could limit adverse drug reactions and improve QoL. This might explain the improvement in adverse effects of other drugs reported by 10.4% of current medical users, who also reported significantly more concomitant medications compared to current recreational users.

Current users and current medical users generally had a daily to three times weekly to daily cannabis consumption, consistent to a chronic medication use, while former users and current recreational users had a more sporadic use. However, only a minority of patients reported an increased consumption over time, altogether suggesting a limited risk of developing pharmacological tolerance or cannabis use disorder. This hypothesis is supported by other reports on the topic [23], however, it should be thoroughly investigated in future dedicated studies. COVID-19 generally led to decreased (mainly due to supply difficulty) or unvaried cannabis consume. For those reporting increased use, the observation that current medical users more frequently reported coping difficulty as a reason for increase compared to current recreational users further support the role of cannabis as a medication in this subset of patients.

Patients more frequently assumed high-THC cannabis, with main sources of supply coming from friends, family, and the street market. Indeed, about a third of current and former users reported some variability of cannabis effects, more frequently unexpected or increased effects, which may be explained by the extremely variable and increasing THC concentration in cannabis available in the illegal market [20]. This could be a safety issue of illegal cannabis self-medication due to inconstant levels of active compounds, leading both to unstable efficacy and dose-related adverse reactions. The more frequent alcohol and tobacco smoke in current users might be related to younger age and, from our data, cannabis consumption did not seem relevantly associated with other psychoactive substances utilization, which was minimal. An association with tobacco smoke was previously reported in UK and Denmark and might be due to smoked cannabis was the primary way of administration [10, 14].

About 60% of current and current medical users reported cannabis-related adverse effects, the most frequent being those commonly expected with cannabinoids use. Memory disturbances were more frequently reported by current users; however, this observation is of difficult interpretation since it could be related to MS severity or being merely casual, since the difference in reporting was non-significant between current medical and recreational users. Legal issues were reported in about 10% of current users, constituting a potential harmful consequence of illegal unprescribed cannabis use in patients, possibly impacting on QoL. In fact, in Italy non-prescribed users could face administrative sanctions if found in possession of small cannabis amounts (e.g., driving license temporary suspension, or provisional prohibition to leave the country): such sanctions could be avoided if the subject follows a therapeutical program.

### Limitations

This study is limited by the cross-sectional design, not allowing for a causal relationship analysis between observed associations. The study was conducted mainly in patients from northern Italy, thus limiting its generalizability to the rest of the Country; future National studies should be encouraged. Even though this may be a limitation, we believe that these data are valuable also for comparisons with results from other nations, since the legal status of cannabis is very different worldwide and indirect comparisons of unprescribed cannabis use in MS may provide further understanding of the topic. Potential duplicates were identified on the basis of similarities of a panel of collected variables since the survey was completely anonymous, possibly misidentifying different patients as duplicates. However, the number of duplicates was limited and we believe that granting the complete anonymity of participants could have led to more sincere responses, improving the reliability of the results. The EDSS was self-reported in terms that patients were instructed in the questionnaire to insert their latest EDSS reported in an outpatient or inpatient visit, if they were able. Given the anonymous design, it was not possible to collect the actual investigator-assessed EDSS. In the design phase, the possibility patients could not correctly report or retrieve their EDSS was considered. Thus, we included the PDDS, which is a patient-reported outcome with a good comparability with the EDSS. Indeed, in our study EDSS and PDDS results were comparable in terms of median levels of disability. Addiction-risk evaluation was performed with simple questions on dose variation and other proxy outcomes. Nevertheless, this was a secondary outcome and specifically designed studies should be performed to evaluate cannabis addiction potential in MS. The classification of MS phenotypes might be imprecise since SPMS is often underdiagnosed and patients might be unable to correctly identify their phenotype. For possible therapeutic effects of unprescribed cannabis, potential recall bias could not be excluded due to the study design. However, this bias should have been limited in the current users subgroup analysis, since it was referred to the year before survey completion. Lastly, we did not meet the expected response rate, leading to a slightly underpowered study. This might be due to the length of the questionnaire. Also, it could not be excluded that elderly patients had limited access to digital devices to complete the online survey, being another potential cause of reduced response rate or selection bias. We did took into account this possibility in the design phase of the study; however, we decided to use a fully anonymous online questionnaire to let patients free to give their most honest responses (i.e., low-reporting bias) as a tradeoff with potential limited selection bias. Still, our study has a considerable sample size, being one of the largest among studies on the topic, which we believe is an added value.

## Conclusion

Unprescribed cannabis use is common in patients with MS in Italy, with prevalence seemingly superior to the general population, often intended for medical use and without the disclosure to the treating physician. Young age, being male, and a free marital status were associated with current use, while higher disability, spasticity and pain, anxiety, reduced QoL, concomitant neurological/psychiatric drugs, and analgesics were associated with current medical users. Unprescribed cannabis appeared relatively safe, with limited addiction risk, and with reported potential benefits other than current indication, with a reduction/discontinuation of medications for anxiety, sleep, pain, depression, or other conditions. These results could be valuable to improve patient-clinician therapeutic alliance and risk assessment of cannabinoids consumption in MS, as well as for policy makers.

## Supplementary Information

Below is the link to the electronic supplementary material.

Supplementary file1 (PDF 1102 KB)

## Data Availability

The data that support the findings of this study are available from the corresponding author upon reasonable request.
