# Correction to: Unprescribed cannabinoids and multiple sclerosis: a multicenter, cross-sectional, epidemiological study in Lombardy, Italy

**DOI:** 10.1007/s00415-024-12560-5

**Published:** 2024-09-03

**Authors:** Riccardo Giossi, Martina Mercenari, Massimo Filippi, Chiara Zanetta, Carlo Giuseppe Antozzi, Laura Brambilla, Paolo Confalonieri, Sebastiano Giuseppe Crisafulli, Eugenia Tomas Roldan, Pietro Annovazzi, Marta Zaffira Conti, Caterina Barrilà, Marco Ronzoni, Monica Grobberio, Attilio Negri, Stefan Gustavsen, Valentina Torri Clerici

**Affiliations:** 1 Neuroimmunology and Neuromuscular Diseases Unit, Fondazione IRCCS Istituto Neurologico Carlo Besta, Via Celoria, 11, 20133 Milan, Italy; 2 Poison Control Center and Clinical Pharmacology Unit, ASST Grande Ospedale Metropolitano Niguarda, Piazza Ospedale Maggiore, 3, 20162 Milan, Italy; 3 Neurology Department, School of Medicine and Surgery and Milan Centre for Neuroscience (NeuroMI), University of Milano-Bicocca, Milan, Italy; 4 Neurology Unit and MS Center, IRCCS San Raffaele Scientific Institute, Milan, Italy; 5 Neuroimaging Research Unit, Division of Neuroscience, IRCCS San Raffaele Scientific Institute, Milan, Italy; 6 Vita-Salute San Raffaele University, Milan, Italy; 7 Neurorehabilitation Unit, IRCCS San Raffaele Scientific Institute, Milan, Italy; 8 Neurophysiology Service, IRCCS San Raffaele Scientific Institute, Milan, Italy; 9 Neurology Unit, Neurorehabilitation Unit, and Multiple Sclerosis Center, Division of Neuroscience, IRCCS San Raffaele Scientific Institute, Via Olgettina, 60, 20132 Milan, Italy; 10 Multiple Sclerosis Center, Neurology II Unit, ASST Valle Olona, Gallarate Hospital, Gallarate, Italy; 11 Department of Neurology, ASST Papa Giovanni XXIII, Bergamo, Italy; 12 Neurology Unit, ASST Rhodense, Ospedale Garbagnate Milanese, Garbagnate Milanese, Italy; 13 Clinical Neuropsychology Lab, Neurology Department and Clinical Psychology Unit, ASST Lariana, Como, Italy; 14 SC SerD Territoriale-SS SerD Boifava, ASST Santi Paolo e Carlo, Milan, Italy; 15 Department of Neurology, Danish Multiple Sclerosis Center, Copenhagen University Hospital-Rigshospitalet, Glostrup, Denmark

**Correction to: Journal of Neurology** 10.1007/s00415-024-12472-4

In the original version of this article, affiliation details for author Riccardo Giossi were incorrectly given as

^2^Poison Control Center and Clinical Pharmacology Unit, ASST Grande Ospedale Metropolitano Niguarda, Piazza Ospedale Maggiore, 3, 20162 Milan, Italy

but should have been

^1^Neuroimmunology and Neuromuscular Diseases Unit, Fondazione IRCCS Istituto Neurologico Carlo Besta, Via Celoria, 11, 20133 Milan, Italy and

^2^Poison Control Center and Clinical Pharmacology Unit, ASST Grande Ospedale Metropolitano Niguarda, Piazza Ospedale Maggiore, 3, 20162 Milan, Italy

The original article has been corrected.

