# Supplementary material for: Unprescribed cannabinoids and multiple sclerosis: a multicenter, cross-sectional, epidemiological study in Lombardy, Italy
Source: J Neurol. 2024 Jun 7;271(11):7186–205. doi: 10.1007/s00415-024-12472-4 (PMC11561032; doi:10.1007/s00415-024-12472-4)
Supplement: Supplementary file 1 — Supplementary file1 (PDF 1102 KB) [file 415_2024_12472_MOESM1_ESM.pdf]

Unprescribed cannabinoids and multiple sclerosis: a multicenter, cross-sectional, epidemiological study in Lombardy, Italy

Authors: Riccardo Giossi, Martina Mercenari, Massimo Filippi, Chiara Zanetta, Carlo Giuseppe Antozzi, Laura Brambilla, Paolo Confalonieri, Sebastiano Giuseppe Crisafulli, Eugenia Tomas Roldan, Pietro Annovazzi, Marta Zaffira Conti, Caterina Barrilà, Marco Ronzoni, Monica Grobberio, Attilio Negri, Stefan Gustavsen, Valentina Torri Clerici

SUPPLEMENTARY MATERIALS

**Page 2.**	**Supplementary Material S1. Participating Centers and personnel**
**Page 3.**	**Supplementary Material S2. STROBE Checklist**
**Page 5.**	**Table S1. Main sociodemographic and clinical characteristics**
**Page 6.**	**Table S2. Description of individual DMT received by included patients**
**Page 7.**	**Table S3. Main sociodemographic and clinical characteristics in RRMS patients**
**Page 9.**	**Table S4. Unprescribed cannabis consumption motivation in RRMS patients**
**Page 10.**	**Table S5. Other sociodemographic variables in RRMS patients**
**Page 11.**	**Table S6. Concomitant medications and other substances in RRMS patients**
**Page 12.**	**Table S7. Cannabis use characteristics in RRMS patients**
**Page 14.**	**Table S8. Cannabis effects on symptoms and medications modification in RRMS patients**

Supplementary Material S1. Participating Centers and personnel

- Fondazione IRCCS Istituto Neurologico Carlo Besta, Milano (*coordinating center*): Riccardo Giossi, Valentina Torri Clerici, Martina Mercenari, Paolo Confalonieri, Laura Brambilla, Carlo Antozzi, Sebastiano Giuseppe Crisafulli, Eugenia Tomas Roldan
- IRCCS Ospedale San Raffaele, Milano: Massimo Filippi, Chiara Zanetta
- ASST Rhodense, Ospedale di Garbagnate Milanese, Garbagnate Milanese: Marco Ronzoni, Caterina Barrilà
- ASST Ospedale Papa Giovanni XXIII, Bergamo: Marta Zaffira Conti
- ASST Valle Olona, PO di Gallarate, Varese: Pietro Annovazzi
- ASST Lariana, Ospedale Sant'Anna, San Fermo della Battaglia, Como: Monica Grobberio

Supplementary Material S2. STROBE Checklist

	Item No	Recommendation	Page
**Title and abstract**	1	(*a*) Indicate the study's design with a commonly used term in the title or the abstract	1
		(*b*) Provide in the abstract an informative and balanced summary of what was done and what was found	2
**Introduction**			
Background/rationale	2	Explain the scientific background and rationale for the investigation being reported	5
Objectives	3	State specific objectives, including any prespecified hypotheses	5
**Methods**			
Study design	4	Present key elements of study design early in the paper	5
Setting	5	Describe the setting, locations, and relevant dates, including periods of recruitment, exposure, follow-up, and data collection	5-6
Participants	6	(*a*) Give the eligibility criteria, and the sources and methods of selection of participants. Describe methods of follow-up	5
		(*b*) For matched studies, give matching criteria and number of exposed and unexposed	5-6
Variables	7	Clearly define all outcomes, exposures, predictors, potential confounders, and effect modifiers. Give diagnostic criteria, if applicable	5-6
Data sources/ measurement	8*	For each variable of interest, give sources of data and details of methods of assessment (measurement). Describe comparability of assessment methods if there is more than one group	5-6
Bias	9	Describe any efforts to address potential sources of bias	NA
Study size	10	Explain how the study size was arrived at	5-6
Quantitative variables	11	Explain how quantitative variables were handled in the analyses. If applicable, describe which groupings were chosen and why	6
Statistical methods	12	(*a*) Describe all statistical methods, including those used to control for confounding	6
		(*b*) Describe any methods used to examine subgroups and interactions	6
		(*c*) Explain how missing data were addressed	6
		(*d*) If applicable, explain how loss to follow-up was addressed	NA
		(*e*) Describe any sensitivity analyses	NA
**Results**			
Participants	13*	(a) Report numbers of individuals at each stage of study—eg numbers potentially eligible, examined for eligibility, confirmed eligible, included in the study, completing follow-up, and analysed	7
		(b) Give reasons for non-participation at each stage	7

		(c) Consider use of a flow diagram	Fig.1
Descriptive data	14*	(a) Give characteristics of study participants (eg demographic, clinical, social) and information on exposures and potential confounders	7-9, Tab.1-6, Suppl.
		(b) Indicate number of participants with missing data for each variable of interest	7-9, Tab.1-6
		(c) Summarise follow-up time (eg, average and total amount)	NA
Outcome data	15*	Report numbers of outcome events or summary measures over time	7-9, Tab.1-6
Main results	16	(*a*) Give unadjusted estimates and, if applicable, confounder-adjusted estimates and their precision (eg, 95% confidence interval). Make clear which confounders were adjusted for and why they were included	7-9, Tab.1-6
		(*b*) Report category boundaries when continuous variables were categorized	7-9, Tab.1-6
		(*c*) If relevant, consider translating estimates of relative risk into absolute risk for a meaningful time period	NA
Other analyses	17	Report other analyses done—eg analyses of subgroups and interactions, and sensitivity analyses	7-10, Tab.1-6, Suppl.
**Discussion**			
Key results	18	Summarise key results with reference to study objectives	10-11
Limitations	19	Discuss limitations of the study, taking into account sources of potential bias or imprecision. Discuss both direction and magnitude of any potential bias	11
Interpretation	20	Give a cautious overall interpretation of results considering objectives, limitations, multiplicity of analyses, results from similar studies, and other relevant evidence	9-11
Generalisability	21	Discuss the generalisability (external validity) of the study results	9-12
**Other information**			
Funding	22	Give the source of funding and the role of the funders for the present study and, if applicable, for the original study on which the present article is based	4

Table S1. Main sociodemographic and clinical characteristics								
**Variable**	**Total population (n=2024)**	**Current user (n=313)**	**Former user (n=304)**	**Never user (n=1407)**	**p-value**	**Current medical user (n=154)**	**Current recreational user (n=159)**	**p-value**
**Nationality, n (%)**					0.901			1.000
Italian	1969 (97.3)	306 (97.8)	296 (97.4)	1367 (97.2)		151 (98.1)	155 (97.5)	
EU	31 (1.5)	5 (1.6)	4 (1.3)	22 (1.6)		2 (1.3)	3 (1.9)	
Extra EU	24 (1.2)	2 (0.6)	4 (1.3)	18 (1.3)		1 (0.7)	1 (0.6)	
**Region of living, n (%)**					0.768			0.719
Lombardy	1584 (78.3)	237 (75.7)	249 (81.9)	1098 (78.4)		109 (70.8)	128 (80.5)	
Piedmont	108 (5.34)	16 (5.1)	15 (4.9)	77 (5.5)		8 (5.2)	8 (5.0)	
Emilia Romagna	52 (2.6)	8 (2.6)	9 (3.0)	35 (2.5)		4 (2.6)	4 (2.5)	
Puglia	38 (1.9)	9 (2.9)	6 (2.0)	23 (1.6)		5 (3.3)	4 (2.5)	
Sicily	36 (1.8)	5 (1.6)	6 (2.0)	25 (1.8)		2 (1.3)	3 (1.9)	
Calabria	33 (1.6)	7 (2.2)	1 (0.3)	25 (1.8)		6 (3.9)	1 (0.6)	
Tuscany	28 (1.4)	6 (1.9)	5 (1.6)	17 (1.2)		4 (2.6)	2 (1.3)	
Campania	26 (1.3)	5 (1.6)	4 (1.3)	17 (1.2)		3 (2.0)	2 (1.3)	
Liguria	26 (1.3)	3 (1.0)	2 (0.7)	21 (1.5)		1 (0.7)	2 (1.3)	
Veneto	25 (1.2)	4 (1.3)	1 (0.3)	20 (1.4)		3 (2.0)	1 (0.6)	
Marche	12 (0.6)	2 (0.6)	3 (1.0)	7 (0.5)		2 (1.3)	0 (0.0)	
Sardinia	11 (1.5)	3 (1.0)	1 (0.3)	7 (0.5)		2 (1.3)	1 (0.6)	
Trentino Alto Adige	8 (0.4)	0 (0.0)	0 (0.0)	8 (0.6)		0 (0.0)	0 (0.0)	
Friuli Venezia Giulia	7 (0.4)	1 (0.3)	0 (0.0)	6 (0.4)		1 (0.7)	0 (0.0)	
Lazio	6 (0.3)	0 (0.0)	1 (0.3)	5 (0.4)		0 (0.0)	0 (0.0)	
Umbria	5 (0.3)	1 (0.3)	0 (0.0)	4 (0.3)		1 (0.7)	0 (0.0)	
Abruzzo	5 (0.3)	2 (0.6)	0 (0.0)	3 (0.2)		1 (0.7)	1 (0.6)	
Basilicata	4 (0.2)	1 (0.3)	1 (0.3)	2 (0.1)		1 (0.7)	0 (0.0)	
Valle d’Aosta	1 (0.1)	1 (0.3)	0 (0.0)	0 (0.0)		0 (0.0)	1 (0.6)	
Molise	1 (0.1)	0 (0.0)	0 (0.0)	1 (0.1)		0 (0.0)	0 (0.0)	
Not living in Italy	8 (0.4)	2 (0.6)	0 (0.0)	6 (0.4)		1 (0.7)	1 (0.6)	
Abbreviations: EU=European union.								

Table S2. Description of individual DMT received by included patients								
**Variable**	**Total population (n=2024)**	**Current user (n=313)**	**Former user (n=304)**	**Never user (n=1407)**	**p-value**	**Current medical user (n=154)**	**Current recreational user (n=159)**	**p-value**
**DMT, n (%)**					-			-
Glatiramer acetate	178 (8.8)	12 (3.8)	24 (7.9)	158 (11.9)		4 (2.6)	8 (5.0)	
Interferon beta-1a	114 (5.6)	9 (2.9)	13 (4.5)	92 (6.5)		5 (3.3)	4 (2.5)	
Interferon beta-1b	16 (0.8)	1 (0.3)	2 (0.7)	13 (1.0)		0	1 (0.6)	
Peginterferon	48 (2.4)	6 (1.9)	5 (1.6)	37 (2.6)		5 (3.3)	1 (0.6)	
Dimethyl fumarate	404 (20.0)	75 (24.0)	69 (22.7)	260 (18.5)		29 (18.8)	46 (28.9)	
Teriflunomide	238 (11.8)	38 (12.1)	25 (8.2)	175 (12.4)		17 (11.0)	21 (13.2)	
Fingolimod	317 (15.7)	48 (15.3)	43 (14.1)	226 (16.1)		25 (16.2)	23 (14.5)	
Siponimod	12 (0.6)	0 (0.0)	3 (1.0)	9 (0.6)		0 (0.0)	0 (0.0)	
Ozanimod	17 (0.8)	2 (0.6)	4 (1.3)	11 (0.8)		1 (0.7)	1 (0.6)	
Ponesimod	2 (0.1)	1 (0.3)	0 (0.0)	1 (0.1)		1 (0.7)	0 (0.0)	
Cladribine	54 (2.7)	14 (4.5)	9 (3.0)	31 (2.2)		5 (3.3)	9 (5.7)	
Natalizumab	188 (9.3)	35 (11.2)	36 (11.8)	117 (8.3)		20 (13.0)	15 (9.4)	
Ocrelizumab	186 (9.2)	36 (11.5)	31 (10.2)	119 (8.5)		20 (13.0)	16 (10.0)	
Ofatumumab	5 (0.3)	1 (0.3)	0 (0.0)	4 (0.3)		0 (0.0)	1 (0.6)	
Rituximab	4 (0.2)	0 (0.0)	0 (0.0)	4 (0.3)		0 (0.0)	0 (0.0)	
Alemtuzumab	21 (1.0)	4 (1.3)	7 (2.3)	10 (0.7)		3 (2.0)	1 (0.6)	
Other immunosuppressants	6 (0.3)	0 (0.0)	1 (0.3)	5 (0.4)		0 (0.0)	0 (0.0)	
Investigational drug in clinical trial	26 (1.3)	6 (1.9)	3 (1.0)	17 (1.2)		4 (2.6)	2 (1.3)	
None	188 (9.3)	25 (8.0)	29 (9.5)	134 (9.5)		15 (9.7)	10 (6.3)	
Abbreviations: DMT=disease-modifying treatment.								

Table S3. Main sociodemographic and clinical characteristics in RRMS patients								
**Variable**	**Total population (n=1564)**	**Current user (n=241)**	**Former user (n=246)**	**Never user (n=1077)**	**p-value**	**Current medical user (n=115)**	**Current recreational user (n=126)**	**p-value**
**Age, years**					**0.0001**			0.6757
Mean (SD)	43.9 (10.9)	37.8 (9.5)	40.5 (9.1)	46.1 (10.8)		38.3 (10.2)	37.3 (8.8)	
Median (IQR)	44 (35-52)	36 (31-44)	40 (34-46)	47 (38-54)		36 (31-45)	36 (31-44)	
**Gender, n (%)**					**<0.001**			0.195
Female	1045 (66.8)	117 (48.6)	141 (57.3)	787 (73.1)		62 (53.9)	55 (43.6)	
Non binary	1 (0.1)	0 (0.0)	0 (0.0)	1 (0.1)		0 (0.0)	0 (0.0)	
Prefer not to answer	2 (0.1)	1 (0.4)	0 (0.0)	1 (0.1)		0 (0.0)	1 (0.8)	
**MS duration from onset, years**					**0.0001**			0.4060
Mean (SD)	13.4 (9.0)	10.4 (7.7)	12.5 (8.3)	14.3 (9.3)		10.3 (8.2)	10.5 (7.1)	
Median (IQR)	12 (6-19)	8 (5-14)	11 (7-17)	13 (7-21)		8 (4-12)	9 (5-14)	
**MS duration from diagnosis, years**					**0.0001**			0.6160
Mean (SD)	11.6 (8.4)	9.0 (7.2)	10.6 (7.4)	12.4 (8.7)		9.1 (6.9)	8.8 (7.5)	
Median (IQR)	10 (5-16)	7 (4-12)	9 (5-14)	11 (5-18)		7 (4-13)	7 (3-12)	
**Patients aware of their EDSS score, n (%)**	608 (38.9)	80 (33.2)	102 (41.5)	426 (39.6)	0.124	49 (42.6)		
**EDSS, points, median (IQR)**	2.0 (1.0-3.0)	2.0 (1.5-3.5)	1.5 (1.0-3.0)	2.0 (1.0-3.0)	0.1232	2.5 (1.5-3.5)	1.5 (1.0-2.5)	**0.004**
**PDDS, points, median (IQR)**	0 (0-2)	0 (0-2)	0 (0-2)	0 (0-2)	0.3782	1 (0-3)	0 (0-1)	**<0.0001**
**PDDS, from no to moderate disability, n (%)**	1275 (81.5)	198 (82.2)	211 (85.8)	866 (80.4)	0.142	83 (72.2)	115 (91.3)	
**Spasticity, yes, n (%)**	2896 (18.3)	54 (22.4)	36 (14.6)	196 (18.2)	0.085	40 (34.8)	14 (11.1)	**<0.001**
**Spasticity, VAS**					0.7342			0.3293
Mean (SD)	4.8 (2.4)	4.5 (2.6)	4.38 (2.2)	4.9 (2.4)		4.7 (2.7)	4.0 (2.4)	
Median (IQR)	5 (3-7)	5 (2-6)	5 (3-6)	5 (3-7)		5 (3-6.5)	4.5 (2-5)	
**Pain, yes, n (%)**	378 (24.2)	57 (23.7)	52 (21.1)	269 (25.0)	0.438	47 (40.9)	10 (7.9)	**<0.001**
**Pain, VAS**					0.1891			0.0849
Mean (SD)	5.4 (2.0)	5.7 (2.1)	4.9 (2.1)	5.4 (2.0)		5.9 (2.1)	4.7 (1.6)	
Median (IQR)	5 (4-7)	6 (4-7)	5 (3-7)	5 (4-7)		6 (4-7)	5 (3-5)	
**Spasm frequency, n (%)**					0.152			**<0.001**
No	1179 (75.4)	167 (69.3)	195 (79.3)	817 (75.9)		61 (53.0)	106 (84.1)	
Mild spasms induced by stimulation	181 (11.6)	38 (15.8)	23 (9.4)	120 (11.1)		25 (21.7)	13 (10.3)	
Spasms occurring g < 1 times per h	150 (10.0)	26 (10.8)	20 (8.1)	104 (9.7)		20 (17.4)	6 (4.8)	
Spasms occurring > 1 times per h	41 (2.6)	6 (2.5)	8 (3.3)	27 (2.5)		6 (5.2)	0 (0.0)	
Spasms occurring > 10 times per h	13 (0.8)	4 (1.7)	0 (0.0)	9 (0.8)		3 (2.6)	1 (0.8)	
**Bowel or bladder impairment, n (%)**					0.246			**0.007**
No	802 (51.3)	131 (54.4)	131 (53.3)	540 (50.1)		48 (41.7)	83 (65.9)	
Mild hesitation, urgency, or retention	448 (28.6)	61 (25.3)	77 (31.3)	310 (28.8)		37 (32.2)	24 (19.1)	
Moderate hesitation, urgency, or retention or stypsis, and/or rare urinary incontinence	259 (16.6)	38 (15.8)	33 (13.4)	188 (17.5)		24 (20.9)	14 (11.1)	
Frequent urinary incontinence; necessity of intermittent auto-catheterization; necessity of manual aid for bowel evacuation	25 (1.6)	8 (3.3)	2 (0.8)	15 (1.4)		5 (4.4)	3 (2.4)	
Almost constant catheterization	8 (0.5)	1 (0.4)	1 (0.4)	6 (0.6)		0 (0.0)	1)0.8)	
Loss of bladder or bowel function; permanent catheterization	8 (0.5)	2 (0.8)	0 (0.0)	6 (0.6)		1 (0.9)	1 (0.8)	
Loss of bladder and bowel function	14 (0.9)	0 (0.0)	2 (0.8)	12 (1.1)		0 (0.0)	0 (0.0)	

**MSQoL-29, mean (SD)**								
Physical functioning	80.9 (27.7)	83.4 (25.3)	84.1 (25.6)	79.65 (28.5)	**0.0497**	75.0 (28.7)	91.2 (18.6)	**<0.0001**
Pain	78.7 (23.6)	79.1 (23.5)	82.1 (20.8)	77.8 (24.1)	0.1129	68.8 (25.5)	88.7 (16.5)	**<0.0001**
Emotional wellbeing	65.2 (19.1)	65.9 (17.9)	66.5 (18.1)	64.7 (19.6)	0.4055	63.0 (17.9)	68.6 (17.6)	**0.0279**
Energy	49.6 (21.5)	49.7 (20.0)	49.7 (18.9)	49.6 (22.2)	0.9786	44.7 (19.2)	54.5 (19.5)	**0.0007**
Cognitive functioning	68.7 (22.3)	66.9 (24.7)	69.8 (21.4)	68.7 (22.0)	0.6008	61.6 (25.2)	71.8 (23.2)	**0.0014**
Health stress	74.5 (24.1)	76.0 (24.2)	75.4 (21.5)	73.7 (24.7)	0.3859	69.3 (25.2)	82.2 (20.5)	**<0.0001**
Sexual functioning	72.5 (32.2)	74.2 (31.9)	70.6 (32.3)	72.5 (32.3)	0.3662	70.7 (33.9)	77.4 (29.6)	0.1150
Change in health	48.9 (22.4)	52.5 (31.9)	51.1 (32.3)	47.5 (21.7)	**0.0080**	49.8 (26.6)	55.0 (21.3)	0.1287
Social functioning	64.8 (27.3)	64.7 (27.8)	66.3 (25.7)	64.5 (27.5)	0.8072	55.1 (28.4)	73.8 (24.1)	**<0.0001**
Health perception	47.5 (30.2)	49.7 (32.0)	48.0 (29.5)	46.9 (30.0)	0.6206	40.1 (30.6)	58.7 (30.1)	**<0.0001**
Overall QoL	68.8 (13.4)	69.3 (17.6)	70.0 (18.1)	68.5 (18.6)	0.6167	63.7 (17.6)	74.5 (16.0)	**<0.0001**
PCS	64.6 (18.0)	66.6 (17.6)	65.8 (17.0)	63.9 (18.3)	0.1272	60.4 (17.8)	72.4 (15.3)	**<0.0001**
MCS	65.5c (18.3)	65.8 (18.0)	66.9 (16.2)	65.2 (18.8)	0.6470	59.3 (18.6)	71.8 (15.1)	**<0.0001**
**HADS**								
Anxiety, mean (SD)	6.2 (4.2)	5.8 (3.9)	6.4 (4.1)	6.2 (4.3)	0.4068	6.3 (4.1)	5.3 (3.7)	0.0670
Depression, mean (SD)	3.9 (3.5)	3.7 (3.2)	3.6 (3.2)	4.1 (3.7)	0.4188	3.9 (3.4)	3.5 (3.1)	0.4678
Anxiety > 7 points, n (%)	566 (36.2)	80 (33.2)	92 (37.4)	394 (36.6)	0.559	39 (33.9)	41 (32.5)	0.891
Depression > 7 points, n (%)	272 (17.4)	35 (14.5)	38 (15.5)	199 (18.5)	0.233	18 (15.7)	17 (13.5)	0.715

Abbreviations: EDSS=expanded disability status scale; HADS=hospital anxiety and depression scale; IQR=interquartile range; MCS=mental composite score; MS=multiple sclerosis; MSQoL-29=29-questions Multiple Sclerosis Quality of Life; PCS=physical composite score; PDDS=patient determined disease steps; QoL=quality of life; RRMS=relapsing remitting MS; SD=standard deviation; VAS=visual analogue scale.

Table S3 (continued)

Table S4. Unprescribed cannabis consumption motivation in RRMS patients								
**Variable**	**Total population (n=1564)**	**Current user (n=241)**	**Former user (n=246)**	**Never user (n=1077)**	**p-value**	**Current medical user (n=115)**	**Current recreational user (n=126)**	**p-value**
**Disclosure of cannabis use to the physician, yes, n (%)**	-	84 (34.9)	-	-	-	53 (46.1)	31 (24.6)	**0.001**
**Cannabis current use motivation, n (%)**					-			-
Recreational	-	126 (52.3)	-	-		-	-	
Medical	-	38 (15.8)	-	-		-	-	
Both	-	77 (32.0)	-	-		-	-	
**Did the patient used cannabis before the last 12 months (former user), yes, n (%)**	470 (30.1)	224 (93.0)	246 (100.0)	-	**<0.001**	103 (89.6)	121 (96.0)	0.076
**Cannabis former use motivation, n (%)**					**<0.001**			**<0.001**
Recreational	365 (77.7)	146 (65.2)	219 (89.0)	-		31 (30.1)	115 (95.0)	
Medical	25 (5.3)	19 (8.5)	6 (2.4)	-		18 (17.5)	1 (0.8)	
Both	80 (17.0)	59 (26.3)	21 (8.5)	-		54 (52.4)	5 (4.1)	
**Who advice the patient to use cannabis for medical use, n (%)[a]**								
Personal idea	-	76 (66.1)	12 (44.4)	-	**0.037**	76 (66.1)	4 (66.7)	1.000
Friends	-	29 (25.2)	9 (33.3)	-	0.391	29 (25.2)	1 (16.7)	1.000
Family	-	10 (8.7)	6 (22.2)	-	**0.045**	10 (8.7)	1 (16.7)	0.443
Internet	-	33 (28.7)	6 (22.2)	-	0.498	33 (28.7)	0 (0.0)	0.187
Patients' groups	-	25 (21.8)	7 (25.9)	-	0.639	25 (21.4)	0 (0.0)	0.343
Physician	-	15 (13.4)	5 (18.5)	-	0.462	15 (13.0)	1 (16.7)	0.581
Other healthcare workers	-	4 (3.5)	1 (3.7)	-	0.954	4 (3.5)	1 (16.7)	0.228
Other	-	15 (13.0)	5 (18.5)	-	0.462	15 (13.0)	1 (16.7)	0.581
**Cannabis use interruption motivation (multiple choices possible), n (%)**					-			-
Lack of effect	-	-	13 (5.3)	-		-	-	
Adverse effects	-	-	41 (16.7)	-		-	-	
Fear of others judgement	-	-	18 (7.3)	-		-	-	
Financial problems	-	-	8 (3.3)	-		-	-	
Legal problems	-	-	18 (7.3)	-		-	-	
Other	-	-	184 (74.8)	-		-	-	
**Patient would use cannabis if it was legal, n (%)**	-	-	-	427 (39.7)	-	-	-	-
**Reason of cannabis use if it was legal, n (%)**					-			-
Recreational	-	-	-	29 (6.8)		-	-	
Medical	-	-	-	303 (71.0)		-	-	
Both	-	-	-	95 (22.3)		-	-	

Abbreviations: RRMS=relapsing remitting multiple sclerosis.
[a] Analysis performed only on those who reported medical or both medical and recreational use among current and former users.

Table S5. Other sociodemographic variables in RRMS patients								
**Variable**	**Total population (n=1564)**	**Current user (n=241)**	**Former user (n=246)**	**Never user (n=1077)**	**p-value**	**Current medical user (n=115)**	**Current recreational user (n=126)**	**p-value**
**Instruction level, n (%)**					0.145			**0.012**
None	2 (0.1)	0 (0.0)	0 (0.0)	2 (0.2)		0 (0.0)	0 (0.0)	
Elementary school	8 (0.5)	0 (0.0)	1 (0.4)	7 (0.7)		0 (0.0)	0 (0.0)	
Secondary school	164 (10.5)	32 (13.3)	23 (9.4)	109 (10.1)		13 (11.3)	19 (15.1)	
High school diploma	743 (47.5)	110 (45.6)	105 (42.7)	528 (49.0)		64 (55.7)	46 (36.5)	
University	478 (30.6)	64 (26.6)	89 (36.2)	325 (30.2)		28 (24.4)	36 (28.6)	
Master/PhD/Postgraduate specializations	169 (10.8)	35 (14.5)	28 (11.4)	106 (9.8)		10 (8.7)	25 (19.8)	
**Work, n (%)**					**<0.001**			**0.011**
Full time	981 (62.7)	162 (67.2)	173 (70.3)	646 (60.0)		66 (57.4)	96 (76.2)	
Part time	289 (18.5)	38 (15.8)	44 (17.9)	207 (19.2)		22 (19.1)	16 (12.7)	
Unemployed	218 (13.9)	39 (16.2)	23 (9.4)	156 (14.5)		25 (21.7)	14 (11.1)	
Retirement	76 (4.39)	2 (0.8)	6 (2.4)	68 (6.3)		2 (1.7)	0 (0.0)	
**Annual income, n (%)**[a]					0.587			**0.037**
0 – 10000 €	207 (15.2)	38 (17.5)	31 (13.8)	138 (15.0)		23 (20.0)	15 (12.8)	
10000 – 15000 €	201 (14.8)	33 (15.2)	29 (12.9)	13.9 (15.1)		16 (1.9)	17 (14.5)	
15000 – 26000 €	464 (34.1)	77 (35.5)	84 (37.3)	303 (33.0)		40 (34.8)	37 (31.6)	
26000 – 55000 €	364 (26.8)	55 (25.4)	64 (28.4)	245 (26.7)		19 (16.5)	36 (30.8)	
55000 – 75000 €	75 (5.5)	10 (4.6)	11 (4.9)	54 (5.9)		2 (1.7)	8 (6.8)	
75000 – 120000 €	42 (3.1)	2 (0.9)	6 (2.7)	34 (3.7)		0 (0.0)	2 (1.7)	
More than 120000 €	8 (0.6)	2 (0.9)	0 (0.0)	6 (0.7)		0 (0.0)	2 (1.7)	
Prefer not to disclose	203	24	21	158		15	9	
**Number of inhabitants of the municipality of residence, n (%)**[a]					0.225			0.414
Less than 2000	69 (4.7)	9 (4.1)	12 (5.0)	48 (4.7)		6 (5.2)	3 (2.5)	
2000 – 10000	388 (26.2)	51 (23.0)	60 (24.8)	277 (27.2)		28 (24.4)	23 (19.5)	
10000 – 50000	486 (32.8)	63 (28.4)	78 (32.3)	345 (33.9)		29 (25.2)	34 (28.8)	
50000 – 250000	225 (15.2)	41 (18.5)	33 (13.6)	151 (14.8)		17 (14.8)	24 (20.3)	
More than 250000	315 (21.2)	58 (36.1)	59 (24.4)	198 (19.4)		24 (23.1)	34 (28.8)	
Do not know	81	19	4	58		11	8	
**Marital status, n (%)**					**<0.001**			0.450
Free	601 (38.4)	128 (53.1)	122 (49.6)	351 (32.6)		65 (56.5)	63 (50.0)	
Married	841 (53.8)	98 (40.7)	107 (43.5)	636 (59.1)		42 (36.5)	56 (44.4)	
Separated/Divorced	122 (7.8)	15 (6.2)	17 (6.9)	90 (8.4)		7 (5.6)	8 (7.0)	
**Housing situation, n (%)**					**<0.001**			0.647
Living with the partner	498 (31.8)	86 (35.7)	77 (31.3)	335 (31.1)		43 (37.4)	43 (34.1)	
Living with partner and children	551 (35.2)	50 (20.8)	82 (33.3)	419 (38.9)		19 (16.5)	31 (24.6)	
Living with children	75 (4.8)	6 (2.5)	15 (6.1)	54 (5.0)		3 (2.6)	3 (2.4)	
Living with parents or family of origin	195 (12.5)	48 (19.9)	30 (12.2)	117 (10.9)		25 (21.7)	23 (18.3)	
Living alone	245 (15.7)	51 (21.2)	42 (17.1)	152 (14.1)		25 (21.7)	26 (20.6)	

Abbreviations: RRMS=relapsing remitting multiple sclerosis.

[a] Patients who preferred not to disclose the data or were not aware/did not know were excluded from the statistical comparison.

**Table S6. Concomitant medications and other substances in RRMS patients**								
**Variable**	**Total population (n=1564)**	**Current user (n=241)**	**Former user (n=246)**	**Never user (n=1077)**	**p-value**	**Current medical user (n=115)**	**Current recreational user (n=126)**	**p-value**
**DMT lines, n (%)[a]**					0.072			0.188
First line	783 (50.1)	106 (44.0)	108 (44.0)	569 (52.8)		44 (38.3)	62 (49.2)	
Second line	661 (42.3)	115 (47.7)	115 (46.8)	431 (40.0)		60 (52.2)	55 (43.7)	
Other	18 (1.2)	2 (0.8)	3 (1.2)	13 (1.2)		2 (1.7)	0 (0.0)	
None	102 (6.5)	18 (7.5)	20 (8.1)	64 (5.9)		9 (7.8)	9 (7.1)	
**Concomitant neurological/psychiatric drugs, n (%)**	370 (23.7)	60 (24.9)	52 (21.1)	258 (24.0)	0.571	39 (33.9)	21 (16.7)	**0.003**
**Concomitant analgesic drugs, n (%)**	402 (25.7)	53 (22.0)	50 (20.3)	299 (27.8)	**0.020**	38 (33.0)	15 (11.9)	**<0.001**
**Prescription cannabinoids in the last 12 months, n (%)**	63 (4.0)	27 (11.2)	7 (2.9)	29 (2.7)	**<0.001**	25 (21.7)	2 (1.6)	**<0.001**
**Alcohol use, n (%)**					**<0.001**			**0.012**
No	569 (36.4)	43 (17.8)	50 (20.4)	476 (44.2)		28 (24.4)	15 (11.9)	
Daily	114 (7.3)	22 (9.1)	26 (10.6)	66 (6.1)		6 (5.2)	16 (12.7)	
2-3 times weekly	220 (14.1)	50 (20.8)	39 (15.9)	131 (12.2)		17 (14.8)	33 (26.2)	
1 time weekly	296 (19.0)	66 (27.4)	58 (23.7)	172 (16.0)		31 (27.0)	35 (27.8)	
1-2 times monthly	223 (14.3)	46 (19.1)	45 (17.8)	132 (12.3)		24 (20.9)	22 (17.5)	
Less than 1 time monthly	140 (9.0)	14 (5.8)	27 (11.0)	99 (9.2)		9 (7.8)	5 (4.0)	
**Tobacco smoke, n (%)**	428 (27.4)	146 (60.6)	89 (36.3)	193 (17.9)	**<0.001**	69 (60.0)	77 (61.1)	0.895
**Other psychoactive substances use, n (%)[b]**					**<0.001**			1.000
Yes	18 (1.2)	11 (4.7)	3 (1.2)	4 (0.37)		5 (4.5)	6 (4.8)	
Prefer not to disclose	16	6	0	10		4	2	

Abbreviations: DMT=disease-modifying treatment; RRMS=relapsing remitting multiple sclerosis.

[a] First line DMTs included glatiramoids, interferons, dimethyl fumarate, and teriflunomide; second line DMTs included fingolimod, Siponimod, ozanimod, ponesimod, cladribine, natalizumab, ocrelizumab, ofatumumab, rituximab, and alemtuzumab; other included other immunosuppressants and investigational drugs in clinical trial.

[b] Patients who preferred not to disclose the data were excluded from the statistical comparison.

Table S7. Cannabis use characteristics in RRMS patients								
**Variable**	**Total population (n=1564)**	**Current user (n=241)**	**Former user (n=246)**	**Never user (n=1077)**	**p-value**	**Current medical user (n=115)**	**Current recreational user (n=126)**	**p-value**
**Main source of cannabis supply, n (%)**					**<0.003**			0.992
Friends or family	-	95 (59.8)	130 (66.3)	-		38 (58.5	57 (60.6)	
Street market	-	48 (30.2)	63 (32.1)	-		20 (30.8)	28 (29.8)	
Dark web	-	7 (4.4)	0 (0.0)	-		3 (4.6)	4 (4.3)	
Own cultivation	-	9 (5.7)	3 (1.5)	-		4 (6.2)	5 (5.3)	
Prefer not to disclose [a]	-	80	47	-		48	32	
**Issues related to cannabis use, n (%)**					**<0.001**			**0.010**
Legal issues	-	7 (3.2)	5 (2.2)	-		4 (3.9)	3 (2.6)	
Supply issues	-	53 (24.1)	16 (7.0)	-		35 (33.7)	18 (15.5)	
Both legal and supply issues	-	9 (4.1)	4 (1.7)	-		5 (4.8)	4 (3.5)	
None	-	151 (68.6)	205 (89.1)	-		60 (57.7)	91 (78.5)	
Prefer not to disclose [a]	-	21	16	-		11	10	
**Weekly cannabis cost estimate, n (%)**					**<0.001**			0.103
0-5 €	-	68 (36.6)	103 (63.2)	-		25 (29.1)	43 (43.0)	
5-10 €	-	38 (20.4)	33 (20.3)	-		15 (17.4)	23 (23.0)	
10-25 €	-	41 (22.0)	17 (10.4)	-		22 (25.6)	19 (19.0)	
25-50 €	-	24 (12.9)	8 (4.9)	-		13 (15.1)	11 (11.0)	
50-100 €	-	12 (6.5)	0 (0.0)	-		9 (10.5)	3 (3.0)	
More than 100 €	-	3 (1.6)	2 (1.2)	-		2 (2.3)	1 (1.0)	
Do not know [a]	-	41	73	-		20	21	
Prefer not to disclose [a]	-	14	9	-		9	5	
**Principal way of cannabis assumption, n (%)**					**0.001**			**0.002**
Smoked (cigarette)	-	205 (85.1)	229 (93.1)	-		88 (76.5)	117 (92.9)	
Smoked (pipe)	-	0 (0.0)	4 (1.6)	-		0 (0.0)	0 (0.0)	
Vaped (electronic cigarette)	-	2 (0.8)	1 (0.4)	-		0 (0.0)	2 (1.6)	
Vaped (vaporization)	-	8 (3.3)	1 (0.4)	-		5 (4.4)	3 (2.4)	
Infuse	-	9 (3.7)	4 (1.6)	-		9 (7.8)	0 (0.0)	
Cooked (cookies, cakes)	-	1 (0.4)	4 (1.6)	-		1 (0.9)	0 (0.0)	
Ingestion as oil	-	14 (5.8)	2 (0.8)	-		11 (9.6)	3 (2.4)	
Other	-	2 (0.8)	1 (0.4)	-		1 (0.9)	1 (0.8)	
**Frequency of cannabis administration, n (%)**					**<0.001**			**<0.001**
Daily	-	95 (39.4)	20 (8.1)	-		53 (46.1)	42 (33.3)	
5-6 days per week	-	6 (2.5)	4 (1.6)	-		6 (5.2)	0 (0.0)	
3-4 days per week	-	34 (14.1)	19 (7.7)	-		15 (13.0)	19 (15.1)	
1-2 times monthly	-	40 (16.6)	63 (25.6)	-		24 (20.9)	16 (12.7)	
Less than 1 time monthly	-	66 (27.4)	140 (56.9)	-		17 (14.8)	49 (38.9)	
**Type of assumed cannabis, n (%)**					**0.013**			**0.015**
High THC (e.g., hashish, marijuana)	-	176 (77.2)	152 (86.9)	-		78 (70.3)	98 (83.8)	
Only CBD (e.g., cannabis light)	-	52 (22.8)	23 (13.1)	-		33 (29.7)	19 (16.2)	
Not aware [a]	-	13	71	-		4	9	
**Amount of consumed cannabis variation, n (%)**					**<0.001**			**<0.001**
Increased during time	-	18 (7.5)	0 (0.0)	-		10 (8.7)	8 (6.4)	
Reduced during time	-	71 (29.5)	171 (69.8)	-		18 (15.7)	53 (42.1)	

Varying depending on the period	-	60 (24.9)	23 (9.4)	-		38 (33.0)	22 (17.5)	
Unvaried	-	92 (38.2)	51 (20.8)	-		49 (42.56)	43 (34.1)	
**Variability of cannabis effects, n (%)**					0.134			0.351
Yes	-	50 (27.2)	40 (35.4)	-		27 (30.3)	23 (24.2)	
No	-	134 (72.8)	73 (64.6)	-		62 (69.7)	72 (753.8)	
Not know/Not remember [a]	-	57	132	-		26	31	
**Type of variability, n (%)**					0.720			0.283
Increased effects	-	20 (40.0)	13 (32.5)	-		10 (37.0)	10 (43.5)	
Reduced effects	-	9 (18.0)	7 (17.5)	-		7 (25.9)	2 (8.7)	
Unexpected effects	-	21 (42.0)	20 (50.0)	-		10 (37.0)	11 (47.8)	
**Cannabis-related adverse effects, n (%)**		155 (64.3)	154 (62.6)		0.708	62 (53.9)	93 (73.8)	**0.002**
Dry mouth	-	99 (63.9)	76 (49.4)	-	**0.012**	45 (72.6)	54 (58.1)	0.088
Mucosal irritation	-	9 (5.8)	6 (3.9)	-	0.598	2 (3.2)	7 (7.5)	0.317
Weakness	-	20 (12.9)	25 (16.2)	-	0.425	5 (8.1)	15 (16.1)	0.221
Dizziness	-	35 (22.6)	66 (42.9)	-	**<0.001**	17 (27.4)	18 (19.4)	0.247
Tachycardia	-	61 (39.4)	47 (30.5)	-	0.121	22 (35.5)	39 (41.9)	0.503
Movement disorders	-	15 (9.7)	15 (9.7)	-	1.000	4 (6.5)	11 (11.8)	0.406
Anxiety	-	35 (22.6)	46 (29.9)	-	0.156	13 (21.0)	22 (23.7)	0.845
Panic	-	11 (7.1)	18 (11.7)	-	0.178	4 (6.5)	7 (7.5)	1.000
Hallucinations	-	4 (2.6)	9 (5.8)	-	0.170	0 (0.0)	4 (4.3)	0.150
Gastrointestinal disturbances	-	4 (2.6)	4 (2.6)	-	1.000	2 (3.2)	2 (2.2)	1.000
Sedation	-	9 (5.8)	21 (13.6)	-	**0.022**	5 (8.1)	4 (4.3)	0.485
Insomnia	-	6 (3.9)	6 (3.9)	-	1.000	3 (4.8)	3 (3.2)	0.684
Memory disturbances	-	33 (21.3)	18 (11.7)	-	**0.031**	17 (27.4)	16 (17.2)	0.161
Other	-	12 (7.7)	15 (9.7)	-	0.553	2 (3.2)	10 (10.8)	0.125

Abbreviations: CBD=cannabidiol; RRMS=relapsing remitting multiple sclerosis; THC=tetrahydrocannabinol.
[a] Patients who preferred not to disclose the data or were not aware/did not know were excluded from the statistical comparison.

Table S7 (continued)

Table S8. Cannabis effects on symptoms and medications modification in RRMS patients								
**Variable**	**Total population (n=1564)**	**Current user (n=241)**	**Former user (n=246)**	**Never user (n=1077)**	**p-value**	**Current medical user (n=115)**	**Current recreational user (n=126)**	**p-value**
**Any MS-related symptoms improved by cannabis assumption, n (%)**					**<0.001**			**<0.001**
Yes	-	141 (89.8)	36 (37.1)	-		100 (100.0)	41 (71.9)	
Not know [a]	-	84	149	-		15	69	
**Symptoms improved by cannabis assumption, n (%)**								
Pain	-	70 (49.7)	19 (52.8)	-	0.852	59 (59.0)	11 (26.8)	**0.001**
Spams or tremor	-	72 (51.1)	16 (44.4)	-	0.576	57 (57.0)	15 (36.6)	**0.041**
Sleep disturbances	-	91 (64.5)	22 (61.1)	-	0.702	72 (72.0)	19 (46.3)	**0.006**
Bowel or bladder disturbances	-	19 (13.5)	4 (11.1)	-	1.000	13 (13.0)	6 (14.6)	0.790
Appetite	-	34 (24.1)	4 (11.1)	-	0.113	24 (24.0)	10 (24.4)	1.000
Anxiety	-	70 (49.7)	15 (41.7)	-	0.457	51 (51.0)	19 (46.3)	0.711
Mood	-	57 (40.4)	12 (33.3)	-	0.566	42 (42.0)	15 (36.6)	0.577
Coping	-	27 (19.2)	4 (11.1)	-	0.331	18 (18.0)	9 (22.0)	0.640
Nausea, vomiting, and GI disturbances	-	11 (7.8)	1 (2.8)	-	0.464	10 (10.0)	1 (2.4)	0.176
Headache	-	38 (27.0)	11 (30.6)	-	0.680	27 (27.0)	11 (26.8)	1.000
Adverse effects of other drugs	-	12 (8.51)	1 (2.8)	-	0.472	12 (12.0)	0 (0.0)	**0.019**
MS improvement	-	51 (36.2)	10 (27.8)	-	0.433	41 (41.0)	10 (24.4)	0.082
Sensory symptoms	-	51 (36.2)	17 (47.2)	-	0.252	34 (34.0)	17 (41.5)	0.443
Others	-	23 (16.3)	2 (5.6)	-	0.114	16 (16.0)	7 (17.1)	1.000
**Drugs for anxiety, sleep, pain, depression, or other conditions reduced due to cannabis assumption, n (%)**					**0.011**			**<0.001**
Yes, dose reduction of some drugs	-	32 (29.9)	7 (16.7)	-		25 (34.7)	7 (20.0)	
Yes, discontinuation of some drugs	-	40 (37.4)	10 (23.8)	-		35 (48.6)	5 (14.3)	
No, unvaried drugs assumption	-	35 (32.7)	25 (59.5)	-		12 (16.7)	23 (65.7)	
No, never assumed these kinds of drugs [b]	-	91	113	-		29	62	
Not know [b]	-	43	91	-		14	29	

Abbreviations: MS=multiple sclerosis; RRMS=relapsing remitting MS.
[a] Patients who did not know were excluded from the statistical comparison.
[b] Patients who did not know or never assumed these kinds of drugs were excluded from the statistical comparison.
